# PIN1 and CDK1 cooperatively govern pVHL stability and suppressive functions

**DOI:** 10.1038/s41418-023-01128-x

**Published:** 2023-02-23

**Authors:** Jiayi Chen, Mei Li, Yeqing Liu, Tangming Guan, Xiao Yang, Yalei Wen, Yingjie Zhu, Zeyu Xiao, Xiangchun Shen, Haoxing Zhang, Hui Tang, Tongzheng Liu

**Affiliations:** 1 College of Pharmacy/International Cooperative Laboratory of Traditional Chinese Medicine Modernization and Innovative Drug Development of Ministry of Education (MOE) of China, Jinan University, Guangzhou, 510632 P. R. China; 2 Department of Pathology, Sun Yat-sen Memorial Hospital, Sun Yat-sen University, Guangzhou, 510120 P. R. China; 3 The Guangzhou Key Laboratory of Molecular and Functional Imaging for Clinical Translation, The First Affiliated Hospital of Jinan University, Guangzhou, 510632 P. R. China; 4 The State Key Laboratory of Functions and Applications of Medicinal Plants, School of Pharmaceutic Sciences, Guizhou Medical University, University Town, Guiyang City and Guian New District, Guiyang, 550025 P. R. China; 5 Guangdong Provincial Key Laboratory of Genome Stability and Disease Prevention, College of Life Sciences and Oceanography, Shenzhen University, Shenzhen, 518055 P. R. China; 6 Department of Central Laboratory, The First Affiliated Hospital of Jinan University, 510632 Guangzhou, P. R. China; 7 Department of Clinical Laboratory, The Fifth Affiliated Hospital of Jinan University Heyuan Shenhe People’s Hospital, Heyuan, 517000 P. R. China; 8 The State Key Laboratory of Functions and Applications of Medicinal Plants, Guizhou Medical University, Guiyang, 550014 P. R. China

**Keywords:** Tumour-suppressor proteins, Tumour-suppressor proteins

## Abstract

The VHL protein (pVHL) functions as a tumor suppressor by regulating the degradation or activation of protein substrates such as HIF1α and Akt. In human cancers harboring wild-type *VHL*, the aberrant downregulation of pVHL is frequently detected and critically contributes to tumor progression. However, the underlying mechanism by which the stability of pVHL is deregulated in these cancers remains elusive. Here, we identify cyclin-dependent kinase 1 (CDK1) and peptidyl-prolyl cis-trans isomerase NIMA-interacting 1 (PIN1) as two previously uncharacterized regulators of pVHL in multiple types of human cancers harboring wild-type *VHL* including triple-negative breast cancer (TNBC). PIN1 and CDK1 cooperatively modulate the protein turnover of pVHL, thereby conferring tumor growth, chemotherapeutic resistance and metastasis both in vitro and in vivo. Mechanistically, CDK1 directly phosphorylates pVHL at Ser80, which primes the recognition of pVHL by PIN1. PIN1 then binds to phosphorylated pVHL and facilitates the recruitment of the E3 ligase WSB1, therefore targeting pVHL for ubiquitination and degradation. Furthermore, the genetic ablation or pharmacological inhibition of CDK1 by RO-3306 and PIN1 by all-trans retinoic acid (ATRA), the standard care for Acute Promyelocytic Leukemia could markedly suppress tumor growth, metastasis and sensitize cancer cells to chemotherapeutic drugs in a pVHL dependent manner. The histological analyses show that PIN1 and CDK1 are highly expressed in TNBC samples, which negatively correlate with the expression of pVHL. Taken together, our findings reveal the previous unrecognized tumor-promoting function of CDK1/PIN1 axis through destabilizing pVHL and provide the preclinical evidence that targeting CDK1/PIN1 is an appealing strategy in the treatment of multiple cancers with wild-type *VHL*.

## Introduction

Triple-negative breast cancer (TNBC) is a highly aggressive subtype of breast cancer that lacks the expression of estrogen receptor (ER), progesterone receptor (PR) and human epidermal growth factor receptor-2 (HER2) [1]. Due to the specific molecular expression pattern, TNBC has few targeted therapy options and conventional chemotherapies such as cisplatin and paclitaxel are still the standard treatment strategies. However, frequent chemo-resistance and cancer metastasis are main causes of treatment failure in TNBC patients [2, 3]. Therefore, to elucidate key mechanisms underlying chemo-resistance and metastasis in TNBC is urgently needed.

Protein pVHL encoded by *VHL* gene functions as a tumor suppressor by acting as the substrate recognition component of the ubiquitin E3 ligase complex including Elongin B/C, Rbx1 and Cullin2 [4]. Under normoxic conditions, pVHL recognizes EglNs mediated prolylhydroxylated HIF1α and HIF2α and the E3 ligase complex targets these substrates for proteasome dependent degradation [5]. Other substrates of pVHL including ZHX2 [6] and SFMBT1 [7] undergo similar prolyl hydroxylation and are targeted for degradation by pVHL dependent E3 ligase complex as well. pVHL also acts as a signal adaptor to regulate the activation of Akt and NF-κB signaling pathways in an E3 activity independent manner [8, 9].

Inactivation of *VHL* by mutations has been demonstrated to cause to the accumulation and/or activation of HIFs, Akt and other substrates, thereby triggering oncogenic pathways and driving the development of the hereditary von Hippel–Lindau (VHL) disease and certain types of human cancer such as sporadic clear-cell renal cell carcinoma (ccRCC) [5, 10, 11]. Unlike the VHL diseases and ccRCC, emerging studies reported that mutations in *VHL* are rare in lung cancer and hepatocellular carcinoma [12, 13]. However, the study of *VHL* status and expression in breast cancer is limited so far. Here, we found the *VHL* gene in breast cancer is largely wild-type by analyzing cBioPortal database. However, the expression of pVHL was significantly downregulated in TNBC specimens. Furthermore, we demonstrated the therapeutic benefit of pVHL in TNBC. Therefore, the identification of novel targets with actionable therapeutic drugs to specially stabilize pVHL could greatly benefit the clinical outcome of TNBC patients.

Several post-translational regulatory mechanisms of pVHL have been previously reported. CK2-mediated phosphorylation mildly reduced pVHL stability, although the mechanism is not yet defined [14, 15]. pVHL itself also undergoes ubiquitination and degradation. The ubiquitin E3 ligase WD repeat and SOCS box-containing protein 1 (WSB1) was reported to promote melanoma metastasis through targeting pVHL for ubiquitination and degradation [16]. In hepatocellular carcinoma, OTUD6B acts as an adaptor protein to decrease the interaction between pVHL and WSB1, repress the pVHL degradation in an enzymatic activity independent manner [17]. However, the development of inhibitors to directly target WSB1 is a challenge due to difficulties in substrate specificity and complexity of ubiquitination selectivity. Drugs or compounds to stabilize pVHL in cancers are not yet conceivable.

In this study, we reveal the cyclin-dependent kinase 1 (CDK1) and peptidyl-prolyl cis-trans isomerase NIMA-interacting 1 (PIN1) as two previously uncharacterized regulators of pVHL stability in multiple cancer types harboring wild-type *VHL* including TNBC. We aim to elucidate the molecular mechanism by which PIN1 and CDK1 cooperatively modulate pVHL stability, contribute to tumor progression and explore their therapeutic potential in the treatment of cancers with wild-type *VHL* including TNBC.

## Materials and methods

### Cell culture, plasmids and antibodies

Cell lines BT-549, MDA-MB-231, HEK293T, A375, PANC-1, LoVo, A2780, MCF-7, MDA-MB-435, MCF-10A and SK-BR-3 were purchased from ATCC (American Type Culture Collection, Maryland, USA). All cell lines were mycoplasma-free and authenticated by short tandem repeat DNA profiling analysis. *VHL*, PIN1 and CDK1 were cloned into pIRES-EGFP, pCMV-HA, pLV.3-FLAG, pGEX4T-1 and pET28a vectors. All site mutants were generated by site-directed mutagenesis (TOYOBO, Osaka, Japan) and verified by sequencing. PIN1, WSB1 and VHL shRNAs were purchased from Sigma-Aldrich Co. (MO, USA). CDK1 and CDK2 shRNAs were kindly provided by Professor Bo Yang, Zhejiang University (Zhejiang, China). Targeting sequences for PIN1 shRNAs are 5′-CCACCGTCACACAGTATTTAT-3′ and 5′-GCCATTTGAAGACGCCTCGTT-3′, respectively. Sequences for CDK1 shRNA are 5′-GCTGTACTTCGTCTTCTAATT-3′. Sequences for CDK2 shRNA are 5′-ACGGAGCTTGTTATCGCAAAT-3′. Sequences for WSB1 shRNA are 5′-GGAGTTTCTCTCGTATCGTAT-3′. Sequences for VHL shRNA are 5′-CCCTATTAGATACACTTCTTA-3′.

Antibodies against ubiquitin (sc-8017, dilution: 1:500), VHL (sc-135657, dilution: 1:500), CDK7 (sc-7344, dilution: 1:500), CDK2 (sc-53219, dilution: 1:500), WSB1 (sc-393200, dilution: 1:500) and CDK1 (sc-54, dilution: 1:500) were purchased from Santa Cruz Biotechnology, Inc. (CA, USA). Anti-FLAG (F1804, dilution: 1:1000), anti-HA (H3663, dilution: 1:1000) and anti-β-Actin (A1978, dilution: 1:5000) antibodies were purchased from Sigma-Aldrich Co. PIN1 (10495-1-AP, dilution: 1:1000) antibody was purchased from Proteintech Group (IL, USA). Anti-CDK substrate antibody (9477 S, dilution: 1:500), anti-p-Akt (T308) antibody (13038 S, dilution: 1:1000) and anti-Akt (pan) antibody (2920 S, dilution: 1:1000) were purchased from CST (Cell Signaling Technology, MA, USA), Inc. Anti-HIF1α (A300-286A, dilution: 1:500) antibody was purchased from Bethyl Laboratories (TX, USA). Western blotting was performed by using antibodies listed above.

### Coimmunoprecipitation assay

Cells were lysed with NETN buffer in ice for 30 min. Cell lysates were incubated with anti-HA magnetic beads, anti-FLAG affinity gel or S-protein agarose for 2 or 4 h at 4 °C. Following precipitation, pellets were washed 4 times with lysis buffer and then analyzed by immunoblotting.

### Denaturating Ni-NTA pulldown

Cells were transfected with indicated constructs and collected cell pellets were lysed in 8 M urea, 0.1 M NaH_2_PO_4_, 300 mM NaCl and 0.01 M Tris (pH 8.0). Lysates were briefly sonicated to shear DNA and incubated with Ni-NTA agarose beads (Invitrogen, CA, USA) for 2 h at 4 °C. Beads were washed four times with 8 M urea, 0.1 M NaH_2_PO_4_, 300 mM NaCl and 0.01 M Tris (pH 8.0). Input and beads were boiled in loading buffer and subjected to SDS–polyacrylamide gel electrophoresis and immunoblotting.

### Denaturing immunoprecipitation for ubiquitination

Cells were lysed in 100 μL 62.5 mM Tris-HCl (PH 6.8), 2% SDS, 10% glycerol, 20 mM NEM and 1 mM iodoacetamide, boiled for 15 min, diluted 10 times with NETN buffer containing protease inhibitors, 20 mM NEM and 1 mM iodoacetamide and centrifuged to remove cell debris. Cell extracts were subjected to immunoprecipitation and blotted as indicated.

### CCK8 assay

Cells were seeded at a density of 2000 cells/well in 96-well plates. The cell viability in each group was evaluated using a CCK8 assay kit (MCE, NJ, USA). The optical density values at a wavelength of 450 nm were measured using a microplate reader to determine cell viability.

### Cell proliferation assay

MDA-MB-231 (3 × 10^4^) or BT-549 (3 × 10^4^) cells were seeded in 6-well plates, and each group was in 6 wells. Cells for one of 6 wells were digested with 0.25% trypsin at 37 °C the next day. Cell pellets were collected by centrifugation, washed by PBS, re-suspended in PBS and counted in microscope. Likewise, cells for the next 5 days were counted in the similar method.

### GST pulldown assay

Recombinant GST-VHL and His-CDK1 proteins were expressed in *Escherichia coli* strain BL21. GST-VHL protein was purified using Pierce Glutathione agarose. Fusion proteins were mixed for 4 h at 4 °C. Beads were washed four times, and proteins were detected by western blotting.

### In vitro kinase assay

The recombinant GST-VHL WT and S80A mutant protein was expressed in *Escherichia coli* strain BL21 and purified using pierce glutathione agarose. Proteins were then eluted with GSH washing buffer (10 mM GSH and 50 mM Tris-HCl, pH = 8.0) and purified with ultrafiltration tube. GST-VHL WT and GST-VHL SA were incubated with purified CDK1 kinase (Carna Biosciences, Kobe, Japan) in kinase buffer (50 mM Tris-HCl, pH 7.4, 50 mM NaCl, 10 mM MgCl_2_, 10 mM β-glycerophosphate, 1 mM dithiothreitol (DTT), and 100 μM ATP) [18, 19]. The reaction was carried out at 30 °C for 30 min and stopped by the addition of SDS loading buffer. Then analyzed by western blotting.

### Animal studies

Female BALB/C nude mice and NOD-SCID mice (5–6 weeks old) were provided by Jicui Yaokang Biotechnology Co., Ltd., Jiangsu, China and were housed under specific-pathogen-free condition in the Animal Center of Jinan University. Animal sample sizes and experimental settings were determined according to our previous publication [20]. For subcutaneous xenografting, MDA-MB-231 cells (10^6^ cells/mouse) were injected subcutaneously in the flank of female BALB/C nude mice (*n* = 6). For the lung metastasis study, MDA-MB-231 cells (1 × 10^6^) were transfected as indicated and injected into the mammary fat pad of female NOD-SCID mice (*n* = 6). When tumors reached 400 mm^3^ in size, the primary tumors were removed. Animals were randomly allocated to different groups and administrated by Vehicle, ATRA (1.5 mg/kg) [21] or RO-3306 (4 mg/kg) [22] every two days until sacrifice. Cisplatin (2 mg/kg) were administrated for three times weekly after xenografting. Tumor volumes were calculated using the following formula: width2 × length × 0.4 (mm^3^). After the tumors had grown for the designated time, all mice were euthanized. The tumors were harvested and weighted. For the lung metastasis study, mice were sacrificed and number of metastatic lung nodules was counted and quantified after 8 weeks. During data collection and analysis, two independent investigators were blinded to the experiment assignment. All animal experiments were performed in accordance with a protocol approved by the Institutional Animal Care and Use Committee of the Jinan University (20211208-08).

### Statistical analysis

Each assay was independently repeated at least three times. Results were presented as mean ± SD. Statistical analyses were performed using GraphPad Prism software version 9.3. One-way ANOVA analysis and Tukey’s test or *t-test* was used to compare results. Statistical significance was defined as *p < 0.05, **p < 0.01 and ***p < 0.001.

## Results

### Identification of PIN1 as a novel regulator of pVHL

**Fig. 1:**
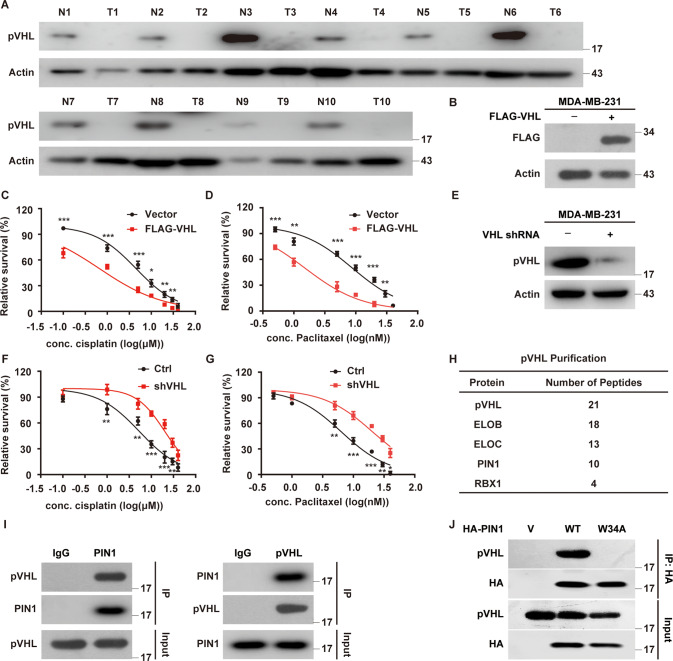
Identification of PIN1 as a novel regulator of pVHL. **A** Western blotting was performed with indicated antibodies in TNBC patient samples and normal breast tissues. **B** Cells stably overexpressing FLAG-VHL were generated. Western blotting was performed. **C**, **D** Cells as in (**B**) were treated with cisplatin or paclitaxel. Cell survival was determined. Results represent the mean ± SD of four independent experiments. *p < 0.05, **p < 0.01, ***p < 0.001, FLAG-VHL vs Vector. **E** Cells stably expressing indicated shRNAs were generated. Western blotting was performed. **F**, **G** Cells as in (**E**) were treated with cisplatin or paclitaxel. Cell survival was determined. Results represent the mean ± SD of four independent experiments. *p < 0.05, **p < 0.01, ***p < 0.001, shVHL vs Ctrl. **H** List of pVHL-associated proteins identified by mass spectrometric analysis. MDA-MB-231 cells stably expressing FLAG-VHL were generated and pVHL complexes were subjected to mass spectrometric analysis. **I** MDA-MB-231 cell lysates were subjected to immunoprecipitation with IgG, anti-PIN1 (left) or anti-pVHL (right) antibodies. Immunoprecipitates were blotted with indicated antibodies. **J** Cell lysates were subjected to immunoprecipitation with anti-HA magnetic beads and western blotting was performed.

Although mutations in *VHL* are frequently detected in VHL disease and ccRCC [5], the status of *VHL* in breast cancer remains poorly defined. We analyzed *VHL* alterations in breast cancers collected in cBioPortal for Cancer Genomics databases and found that *VHL* gene is largely wild-type in breast cancer (Supplementary Fig. 1A). However, the expression of pVHL was significantly lower in TNBC tissues than in normal breast tissues (Fig. 1A and Supplementary Fig. 1B). We also found the expression of pVHL in several TNBC cell lines was lower than that in a human normal mammary epithelial cell line MCF-10A (Supplementary Fig. 1C). Next, we demonstrated the therapeutic benefit of pVHL in TNBC as the over-expression of *VHL* dramatically reduced the proliferation, migration and invasion ability of MDA-MB-231 cells as well as sensitized cells to cisplatin and paclitaxel (Fig. 1B–D and Supplementary Fig. 1D–F). Meanwhile, the knockdown of *VHL* significantly displayed the opposite effects (Fig. 1E–G and Supplementary Fig. 1G–I). These results strongly pointed to the suppressive role of pVHL in TNBC. Furthermore, we examined the clinical relevance of pVHL by analyzing Kaplan-Meier database and found that individuals with high *VHL* expression in TNBC and other cancers harboring wild-type *VHL* such as pancreatic ductal adenocarcinoma and rectum adenocarcinoma, showed higher recurrence-free survival, which is consistent with previous studies [23, 24] (Supplementary Fig. 1J–L).

To identify potential regulators of pVHL in TNBC, we used MDA-MB-231 cells stably expressing FLAG-VHL to perform tandem affinity purification and mass spectrometry analysis. In addition to some known pVHL interacting proteins such as ELOB/C and Rbx1 [25], we identified peptidyl-prolyl cis-trans isomerase NIMA-interacting 1 (PIN1) as a potential pVHL interacting protein (Fig. 1H). We next confirmed the endogenous PIN1-pVHL interaction by coimmunoprecipitation in MDA-MB-231 cells (Fig. 1I). WW domain is the specific region of PIN1 to bind pS/T-P motifs of substrates and the W34A mutation in WW domain could abolish the interaction between PIN1 and its substrates [26]. We also found that PIN1 WT, but not the W34A mutant, coimmunoprecipitated with pVHL (Fig. 1J).

### PIN1 regulates the ubiquitination and degradation of pVHL

**Fig. 2:**
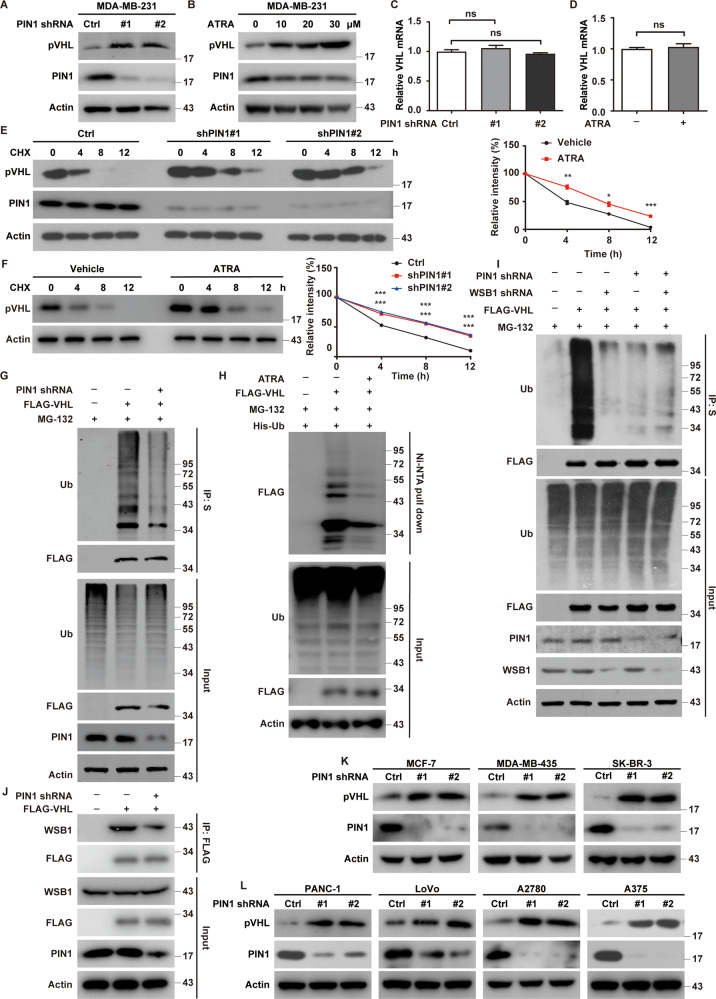
PIN1 regulates the stability of pVHL by affecting its ubiquitination. **A** MDA-MB-231 cells stably expressing control or PIN1 shRNAs were generated and western blotting was performed with indicated antibodies. **B** MDA-MB-231 cells were treated with ATRA and western blotting was performed with indicated antibodies. **C** Total RNA was isolated from cells in (**A**). Relative expression of VHL in cells stably expressing control or PIN1 shRNAs was determined by quantitative PCR. Results represent the mean ± SD of three independent experiments. shPIN1#1 vs Ctrl, shPIN1#2 vs Ctrl. **D** Total RNA was isolated from cells were treated with ATRA. Relative expression of VHL in cells was determined by quantitative PCR. Results represent the mean ± SD of three independent experiments. ATRA vs Vehicle. **E** Cycloheximide pulse-chase assay was performed in cells as in (**A**) and results were quantified (right). Results represent the mean ± SD of three independent experiments. *p < 0.05, **p < 0.01, ***p < 0.001, shPIN1#1 vs Ctrl, shPIN1#2 vs Ctrl. **F** MDA-MB-231 cells were treated with ATRA for 24 h. Cycloheximide pulse-chase assay was performed in cells and results were quantified (right). Results represent the mean ± SD of three independent experiments. ***p < 0.001, ATRA vs Vehicle. **G** HEK293T cells stably expressing control or PIN1 shRNAs were generated and transfected with indicated plasmids and treated with MG-132 for 8 h before cell lysates were immunoprecipitated with S-protein agarose, and the polyubiquitylated pVHL was detected by anti-ubiquitin antibody. **H** Cells were transfected with indicated constructs and treated with Vehicle or ATRA for 24 h in the presence of MG-132. Ubiquitinated proteins were pulled down under denaturing conditions by Ni-NTA agarose and analyzed by immunoblot. **I** HEK293T cells stably expressing control, PIN1 or WSB1 shRNAs were generated and transfected with indicated plasmids and treated with MG-132 for 8 h before cell lysates were immunoprecipitated with S-protein agarose, and the polyubiquitylated pVHL was detected by anti-ubiquitin antibody. **J** Cells were infected with indicated plasmids and cell lysates were subjected to immunoprecipitation with anti-FLAG affinity gel and western blotting was performed. **K**, **L** Cells stably expressing control or PIN1 shRNAs were generated and western blotting was performed with indicated antibodies.

The interaction of PIN1 and pVHL prompted us to examine a potential role for PIN1 in the regulation of pVHL. We found that the knockdown of PIN1 in MDA-MB-231 and BT-549 significantly increased the protein level of pVHL (Fig. 2A and Supplementary Fig. 2A). All-trans retinoic acid (ATRA), approved by FDA for the treatment of Acute Promyelocytic Leukemia (APL) [27], has been reported to be a potent PIN1 inhibitor [28, 29]. Consistently, the treatment of ATRA dose-dependently increased pVHL protein level in MDA-MB-231 and BT-549 cells (Fig. 2B and Supplementary Fig. 2B). The regulation of pVHL by PIN1 was not at the level of transcription since no apparent difference of VHL mRNA levels were detected in control and PIN1 depleted cells (Fig. 2C and Supplementary Fig. 2C). Similar results were also observed in cells treated with ATRA (Fig. 2D). We also found that pVHL was more stable in PIN1-deficient cells than control assessed by cycloheximide pulse-chase assay (Fig. 2E). The inhibition of PIN1 by ATRA also prolonged the half-life of pVHL in MDA-MB-231 cells (Fig. 2F). These results suggest that targeting of PIN1 by the genetic ablation or pharmacological inhibition could dramatically stabilizes pVHL in TNBC.

Since ubiquitin-proteasome system is a main pathway of protein degradation [30], ubiquitination assay were performed to investigate whether PIN1 destabilizes pVHL by affecting its ubiquitination. As showed in Fig. 2G, a significant decrease of polyubiquitylated pVHL was observed in PIN1-deficient cells. Similarly, ATRA treatment also decreased the ubiquitination of pVHL (Fig. 2H and Supplementary Fig. 2D). The E3 ligase WSB1 is reported to target pVHL to ubiquitination and degradation [16]. Next, we examined whether PIN1 promoted pVHL ubiquitination by affecting its interaction with WSB1. As showed in Fig. 2I, the knockdown of PIN1 or WSB1 alone reduced the ubiquitination level of pVHL, while the combination depletion of PIN1 and WSB1 did not further decrease it, indicating PIN1 might regulate the ubiquitination of pVHL in a WSB1-dependent manner. Intriguingly, the depletion of PIN1 or the pharmacological inhibition of PIN1 by ATRA dramatically decreased the interaction between WSB1 and pVHL (Fig. 2J and Supplementary Fig. 2E). These results indicate that PIN1 could promote the WSB1-pVHL interaction, thereby increasing the ubiquitination and proteasome dependent degradation of pVHL in TNBC. Next, we investigated the effect of PIN1 on pVHL in other types of cancers. As showed in Fig. 2K, L, the PIN1-pVHL axis might be a common regulatory mechanism since the depletion of PIN1 significantly increased pVHL levels in various cancer cells including melanoma, pancreatic cancer, colorectal cancer, ovarian cancer and some other subtypes of breast cancers. We also found that pVHL was more stable in ER^+^ (MCF-7) and HER2^+^ (SK-BR-3) breast cancer cell lines when PIN1 was knockdown by specific shRNA, which could be due to the decreased ubiquitination of pVHL (Supplementary Fig. 2F–I). These results highlight the clinical potential of targeting PIN1 towards pVHL stability across various types of cancers harboring wild-type *VHL*.

### PIN1 promotes tumor progression through destabilizing pVHL

**Fig. 3:**
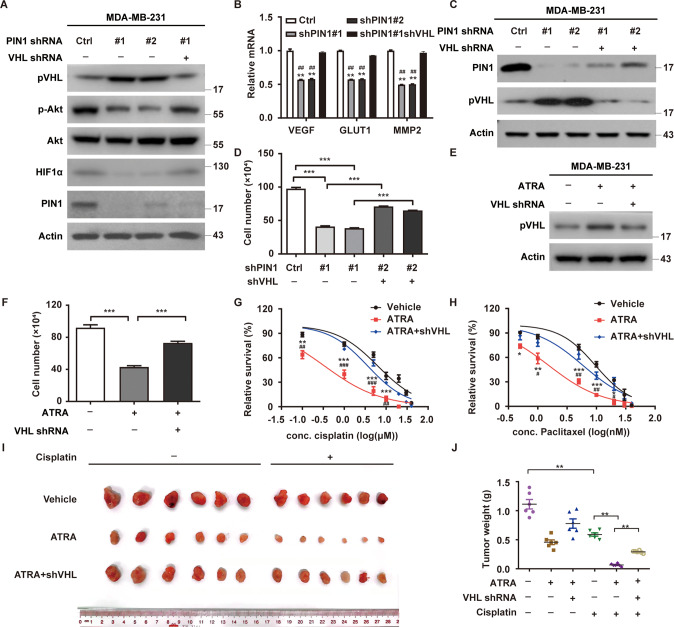
PIN1 promotes tumor progression of TNBC through pVHL. **A** MDA-MB-231 cells stably expressing control, PIN1 or VHL shRNAs were generated and western blotting was performed with indicated antibodies. **B** Total RNA was isolated from MDA-MB-231 cells. Relative expression of VEGF, GLUT1 and MMP2 in cells stably expressing control, PIN1 or VHL shRNAs were determined by quantitative PCR. Results represent the mean ± SD of three independent experiments. **p < 0.01, shPIN1#1 vs Ctrl, shPIN1#2 vs Ctrl. ^##^*p* < 0.01, shPIN1#1shVHL vs shPIN1#1, shPIN1#1shVHL vs shPIN1#2. **C** Cells were infected with indicated shRNAs. Western blotting was performed. **D** Cell proliferation assay was performed in MDA-MB-231 cells. Results represent the mean ± SD of three independent experiments. ***p < 0.001, shPIN1#1 vs Ctrl, shPIN1#2 vs Ctrl, shPIN1#1shVHL vs shPIN1#1, shPIN1#1shVHL vs shPIN1#2. **E** MDA-MB-231 cells were treated with ATRA and western blotting was performed with indicated antibodies. **F** Cell proliferation assay was performed in MDA-MB-231 cells. Results represent the mean ± SD of three independent experiments. ***p < 0.001, ATRA vs Vehicle, ATRA + shVHL vs ATRA. **G**, **H** MDA-MB-231 cells were treated with ATRA. Cell survival was determined. Results represent the mean ± SD of four independent experiments. *p < 0.05, **p < 0.01, ***p < 0.001, ATRA vs Vehicle. ^#^*p* < 0.05, ^##^*p* < 0.01, ^###^*p* < 0.001, ATRA + shVHL vs ATRA. **I**, **J** Mice with subcutaneously established tumors from MDA-MB-231 cells stably expressing indicated shRNAs were treated with Vehicle, ATRA (1.5 mg/kg), cisplatin (2 mg/kg) or combination. Mice were sacrificed to dissect xenograft tumors and measure tumor weights. **J** Results represent the mean ± SD of six independent experiments. **p < 0.01, Vehicle + Cisplatin vs Vehicle, ATRA + Cisplatin vs Vehicle + Cisplatin, ATRA + shVHL + Cisplatin vs ATRA + Cisplatin.

PIN1 has been reported to promote tumor progression of various cancers [31]. We next investigated whether the tumor promoting function of PIN1 in TNBC is mediated by pVHL. As showed in Fig. 3A and Supplementary Fig. 3A, the depletion of PIN1 in MDA-MB-231 and BT-549 cells significantly increased pVHL levels, along with markedly decreased levels of HIF1α and Akt phosphorylation, two known protein substrates of pVHL [8, 32]. Similar results were observed when cells were treated with ATRA (Supplementary Fig. 3B, C). We also found the deficiency of PIN1 dramatically decreased mRNA levels of HIF1α target genes *VEGF*, *GLUT1* and *MMP2* [5], which could be largely restored by the depletion of *VHL* (Fig. 3B and Supplementary Fig. 3D). These results suggest that PIN1 destabilizes pVHL in TNBC, thereby activating its downstream signaling pathways such as HIF1α and Akt.

We next investigated whether PIN1 could promote tumor progression of TNBC through destabilizing pVHL. As showed in Fig. 3C, D and Supplementary Fig. 3E–I, the depletion of PIN1 markedly reduced cell proliferation, migration and invasion of MDA-MB-231 and BT-549 in vitro, which was markedly restored by the depletion of *VHL*. Similar results were observed when cells were treated with ATRA (Fig. 3E, F and Supplementary Fig. 4A–E). Moreover, the treatment of ATRA significantly increased cellular sensitivity to cisplatin and paclitaxel, while the depletion of *VHL* could rescue such an effect (Fig. 3G, H and Supplementary Fig. 4F, G). Consistently, results of xenograft experiments showed that ATRA inhibited tumor growth and increased the sensitivity to cisplatin, which was largely abrogated by the depletion of *VHL* (Fig. 3I, J and Supplementary Fig. 4H). Taken together, these results suggest that PIN1 promotes tumor progression of TNBC both in vitro and in vivo at least in part through affecting the stability of pVHL.

### The phosphorylation of pVHL at Ser80 promotes its ubiquitination and degradation

**Fig. 4:**
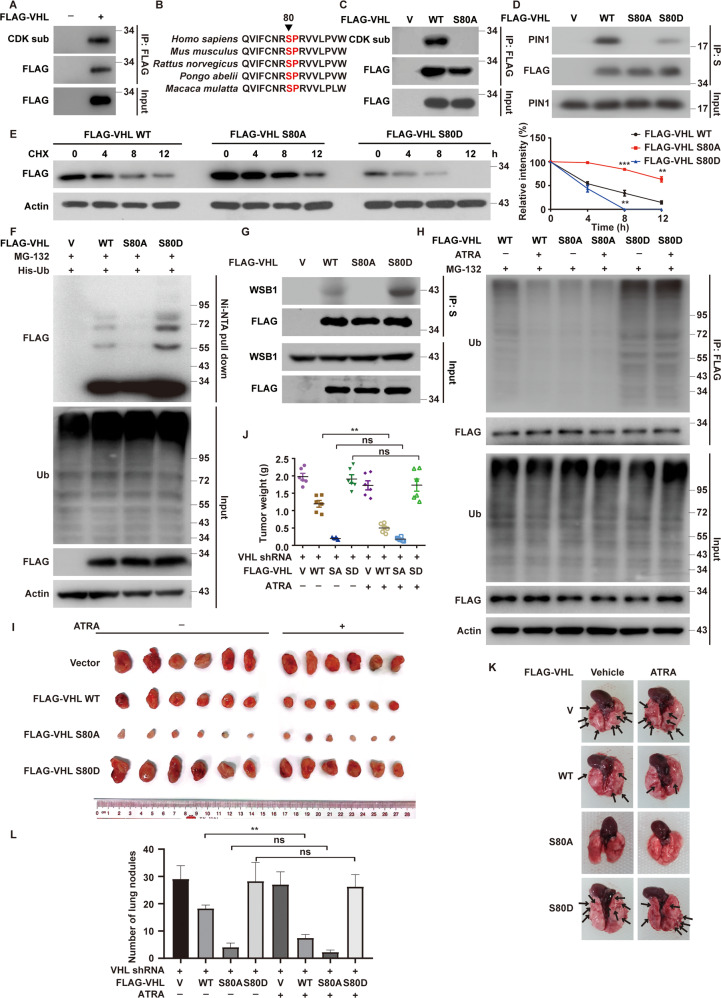
The phosphorylation of pVHL at Ser80 promotes the degradation of pVHL by ubiquitin-proteasome system. **A** Vector or FLAG-VHL was infected in MDA-MB-231 cells. Cell lysates were subjected to immunoprecipitation with anti-FLAG affinity gel and the phosphorylation of pVHL was examined. **B** AA sequences around S80 residue in pVHL are conserved across different species. Arrows, serine residues that are conserved across species. **C** Cell lysates were subjected to immunoprecipitation with anti-FLAG affinity gel and the phosphorylation of pVHL was examined. **D** Cell lysates were subjected to immunoprecipitation with S-protein agarose and western blotting was performed. **E** Cycloheximide pulse-chase assay was performed in MDA-MB-231 cells and results were quantified (right). Results represent the mean ± SD of three independent experiments. **p < 0.01, ***p < 0.001, FLAG-VHL SA vs FLAG-VHL WT, FLAG-VHL SD vs FLAG-VHL WT. **F** Cells were transfected with indicated constructs and treated with MG-132 for 8 h. Ubiquitinated proteins were pulled down under denaturing conditions by Ni-NTA agarose and analyzed by immunoblot. **G** Cells were transfected with indicated plasmids and cell lysates were subjected to immunoprecipitation with S-protein agarose and western blotting was performed. **H** Cells were infected with indicated constructs and treated with Vehicle or ATRA for 24 h in the presence of MG-132. Cell lysates were immunoprecipitated with anti-FLAG affinity gel and the polyubiquitylated pVHL was detected by anti-ubiquitin antibody. **I**, **J** Mice with subcutaneously established tumors from MDA-MB-231 cells stably expressing indicated plasmid were treated with Vehicle or ATRA (1.5 mg/kg). Mice were sacrificed to dissect xenograft tumors and measure tumor weights (**J**). Results represent the mean ± SD of six independent experiments. **p < 0.01, WT + ATRA vs WT + Vehicle, SA + ATRA vs SA + Vehicle, SD + ATRA vs SD + Vehicle. **K**, **L** Cells were injected into mammary fat pad of mice (*n* = 6). After 8 weeks, mice were sacrificed to count and quantify lung metastatic nodules. Arrowheads indicate metastases. Results represent the mean ± SD of six independent experiments. **p < 0.01, WT + ATRA vs WT + Vehicle, SA + ATRA vs SA + Vehicle, SD + ATRA vs SD + Vehicle.

PIN1 binds to the S/TP motif only when the preceding serine or threonine is phosphorylated [31]. We observed the proline-directed phosphorylation of FLAG-VHL by using CDK substrate antibody (Fig. 4A). Interestingly, only one Ser80-Pro81 motif was contained in amino acid sequence of pVHL, which is highly conserved among multiple species (Fig. 4B). As showed in Fig. 4C, the proline-directed phosphorylation of pVHL was completely abrogated by the S80A mutant, indicating that Ser80 is the proline-directed phosphorylation site in pVHL. We also found that pVHL WT but not S80A could interact with PIN1, while the interaction of the S80D mutant and PIN1 is much weaker than WT (Fig. 4D), indicating that phosphorylation of pVHL at Ser80-Pro81 motif is required for pVHL-PIN1 interaction.

We next examined the effect of Ser80 on pVHL ubiquitination and degradation. As showed in Fig. 4E and Supplementary Fig. 5A, the phosphorylation mimic mutant S80D degraded much faster than WT, while the mutant S80A was much more stabilized. Consistently, the ubiquitination levels of S80A were much weaker than WT and S80D (Fig. 4F), which might be due to the abolished interaction between S80A and WSB1 (Fig. 4G). Interestingly, the *VHL* mutation P81S with the gain-function and the low tumorigenicity in patients [33] abolished the interaction between pVHL and WSB1 (Supplementary Fig. 5B). We also found that the treatment of ATRA caused a significant decrease of polyubiquitylated pVHL WT but could not affect the ubiquitination of pVHL S80A or S80D mutant, indicating this phosphorylation event is crucial for the destabilization of pVHL by PIN1 (Fig. 4H).

Next, we determined whether the effect of PIN1 on the suppressive role of pVHL depended on this phosphorylation. As showed in Supplementary Fig. 5C, the reconstitution of S80A in endogenous *VHL*-deficient cells displayed the strongest inhibitory effect on cell proliferation. Interestingly, *VHL* WT inhibited cell proliferation to a lesser extent, which was remarkably enhanced by the treatment of ATRA. Meanwhile, the treatment of ATRA did not cause any significant change in cells reconstituted with the *VHL* S80A or S80D mutant. Similar results were observed in xenograft experiment (Fig. 4I, J and Supplementary Fig. 5D). Regarding the fact that pVHL suppresses cancer metastasis by inhibiting the stability or activity of HIF1α and other substrates [34–36], we next assessed the role of this phosphorylation event in metastasis. As showed in Fig. 4K, L, the reconstitution of WT and S80A significantly inhibited lung colonization compared to S80D, although the inhibitory effect of S80A was much stronger than WT. More interestingly, the treatment of ATRA markedly suppressed lung colonization of cells reconstituted with *VHL* WT in mice, but had no obvious effects on cells reconstituted with *VHL* S80A or S80D (Fig. 4K, L). Together, these results indicate that the phosphorylation of pVHL at Ser80 is pivotal to promote tumor progression through destabilizing pVHL.

### CDK1 binds and phosphorylates pVHL at serine 80

**Fig. 5:**
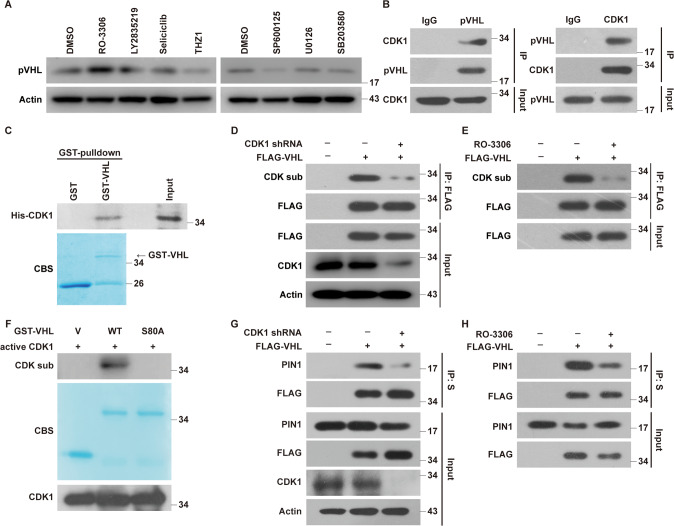
CDK1 directly binds and phosphorylates pVHL. **A** MDA-MB-231 cells were treated with RO-3306 (CDK1 inhibitor), LY2834219 (CDK4/6 inhibitor), Seliciclib (CDK5 inhibitor), THZ1 (CDK7 inhibitor), SP600125 (JNK inhibitor), U0126 (ERK inhibitor) and SB203580 (p38 inhibitor), western blotting was performed with indicated antibodies. **B** MDA-MB-231 cell lysates were subjected to immunoprecipitation with IgG, anti-pVHL (left) or anti-CDK1 (right) antibodies. Immunoprecipitates were blotted with indicated antibodies. **C** Purified recombinant GST, GST-VHL and His-CDK1 were incubated in vitro as indicated. The interaction between pVHL and CDK1 was examined. CBS, Coomassie blue staining. **D** MDA-MB-231 were infected with indicated plasmids and cell lysates were subjected to immunoprecipitation with anti-FLAG affinity gel and western blotting was performed. **E** MDA-MB-231 were infected with indicated plasmids and treated with Vehicle or RO-3306 for 24 h and cell lysates were subjected to immunoprecipitation with anti-FLAG affinity gel and western blotting was performed. **F** Bacterial expressed GST-VHL WT and GST-VHL S80A fusion proteins were incubated with active CDK1 in the presence of ATP. The phosphorylation of pVHL was examined by western blotting. **G** Cells were transfected with indicated plasmids and cell lysates were subjected to immunoprecipitation with S-protein agarose and western blotting was performed. **H** Cells were transfected with indicated plasmids and treated with Vehicle or RO-3306 for 24 h and cell lysates were subjected to immunoprecipitation with S-protein agarose and western blotting was performed.

To identify the specific kinase to phosphorylate pVHL at Ser80, different inhibitors of proline-directed kinases were used to treat MDA-MB-231 cells. We found that CDK1 inhibitor RO-3306 significantly increased pVHL level, while the depletion of CDK2 did not affect it (Fig. 5A and Supplementary Fig. 6A). We next confirmed the interaction between CDK1 and pVHL by coimmunoprecipitation (Fig. 5B and Supplementary Fig. 6B). In addition, purified GST-pVHL protein could bind His-CDK1 in vitro, indicating the direct interaction between pVHL and CDK1 (Fig. 5C). We next assessed the effect of CDK1 on pVHL phosphorylation. As showed in Fig. 5D, E, the phosphorylation of pVHL was markedly reduced by the depletion or the pharmacological inhibition of CDK1. Moreover, the active CDK1 could phosphorylate GST-pVHL WT in vitro, but not the S80A mutant (Fig. 5F). We next examined whether CDK1 could affect the PIN1-pVHL interaction since PIN1 only binds to pSer/Thru-Pro motifs of protein substrates. The depletion or the inhibition of CDK1 by RO-3306 dramatically reduced the interaction between PIN1 and pVHL (Fig. 5G, H). These findings provide the first evidence that CDK1 phosphorylates pVHL at Ser80, which is essential for PIN1-pVHL interaction.

### CDK1 phosphorylates pVHL and promotes its ubiquitination and degradation

**Fig. 6:**
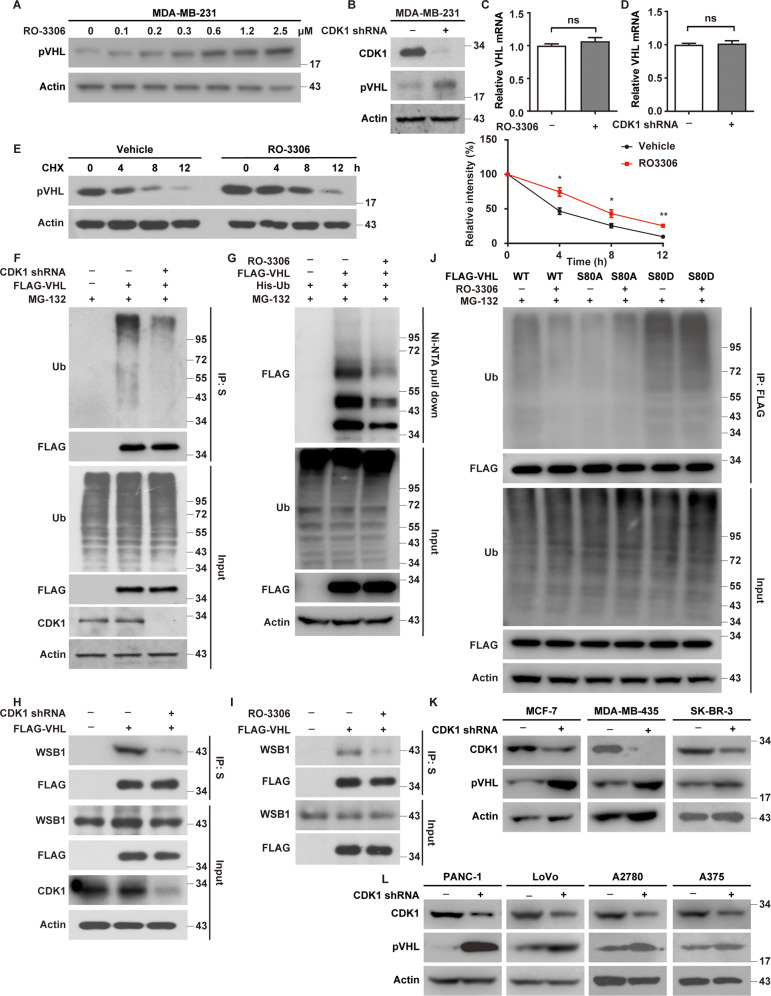
CDK1 phosphorylates pVHL and promotes its degradation by ubiquitin-proteasome system. **A** MDA-MB-231 cells were treated with RO-3306 and western blotting was performed with indicated antibodies. **B** MDA-MB-231 cells were infected with indicated shRNAs. Western blotting was performed. **C** Total RNA was isolated from cells were treated with RO-3306. Relative expression of VHL in cells was determined by quantitative PCR. Results represent the mean ± SD of three independent experiments. RO-3306 vs Vehicle. **D** Total RNA was isolated from cells in (**B**). Relative expression of VHL in cells stably expressing control or CDK1 shRNAs was determined by quantitative PCR. Results represent the mean ± SD of three independent experiments. shCDK1 vs Ctrl. **E** MDA-MB-231 cells were treated with RO-3306 for 24 h. Cycloheximide pulse-chase assay was performed in cells and results were quantified (right). Results represent the mean ± SD of three independent experiments. *p < 0.05, **p < 0.01, RO-3306 vs Vehicle. **F** Cells stably expressing control or CDK1 shRNAs were generated and transfected with indicated plasmids and treated with MG-132 for 8 h before cell lysates were immunoprecipitated with S-protein agarose, and the polyubiquitylated pVHL was detected by anti-ubiquitin antibody. **G** Cells were transfected with indicated constructs and treated with Vehicle or RO-3306 for 24 h in the presence of MG-132. Ubiquitinated proteins were pulled down under denaturing conditions by Ni-NTA agarose and analyzed by immunoblot. **H** Cells were transfected with indicated plasmids and shRNAs. Cell lysates were subjected to immunoprecipitation with S-protein agarose and western blotting was performed. **I** Cells were transfected with indicated plasmids and treated with Vehicle or RO-3306 for 24 h and cell lysates were subjected to immunoprecipitation with S-protein agarose and western blotting was performed. **J** Cells were infected with indicated constructs and treated with Vehicle or RO-3306 for 24 h in the presence of MG-132. Cell lysates were immunoprecipitated with anti-FLAG affinity gel, and the polyubiquitylated pVHL was detected by anti-ubiquitin antibody. **K**, **L** Cells stably expressing control or CDK1 shRNAs were generated and western blotting was performed with indicated antibodies.

Considering the fact that the phosphorylation of pVHL at Ser80 is important for the destabilization of pVHL by PIN1, we hypothesized that CDK1 might destabilize pVHL similarly. As showed in Fig. 6A–D and Supplementary Fig. 7A–C, the depletion or the inhibition of CDK1 could increase the protein level of pVHL in TNBC cells without affecting its mRNA levels. In addition, pVHL was more stable in cells treated with RO-3306 assessed by cycloheximide pulse-chase assay (Fig. 6E and Supplementary Fig. 7D). We next investigate the effect of CDK1 on the ubiquitination of pVHL. As showed in Fig. 6F, G, a significant decrease of polyubiquitylated pVHL was observed in CDK1-deficent cells or in cells treated with RO-3306, which might be caused by the decreased interaction between pVHL and WSB1 (Fig. 6H, I). We further assessed the effect of CDK1 inhibition on the ubiquitination of *VHL* WT, S80D and S80A. As showed in Fig. 6J, the treatment of RO-3306 significantly decreased polyubiquitylated *VHL* WT, but did not affect the ubiquitination of S80A or S80D. More intriguingly, the depletion of CDK1 significantly increased pVHL levels in other types of cancer cells including melanoma, pancreatic cancer, colorectal cancer, ovarian cancer and other kinds of human breast cancers (Fig. 6K, L). Together, these findings provide the first evidence that CDK1 could phosphorylate and destabilize pVHL in multiple cancers with wild-type *VHL*.

### CDK1 promotes tumor progression through destabilizing pVHL

**Fig. 7:**
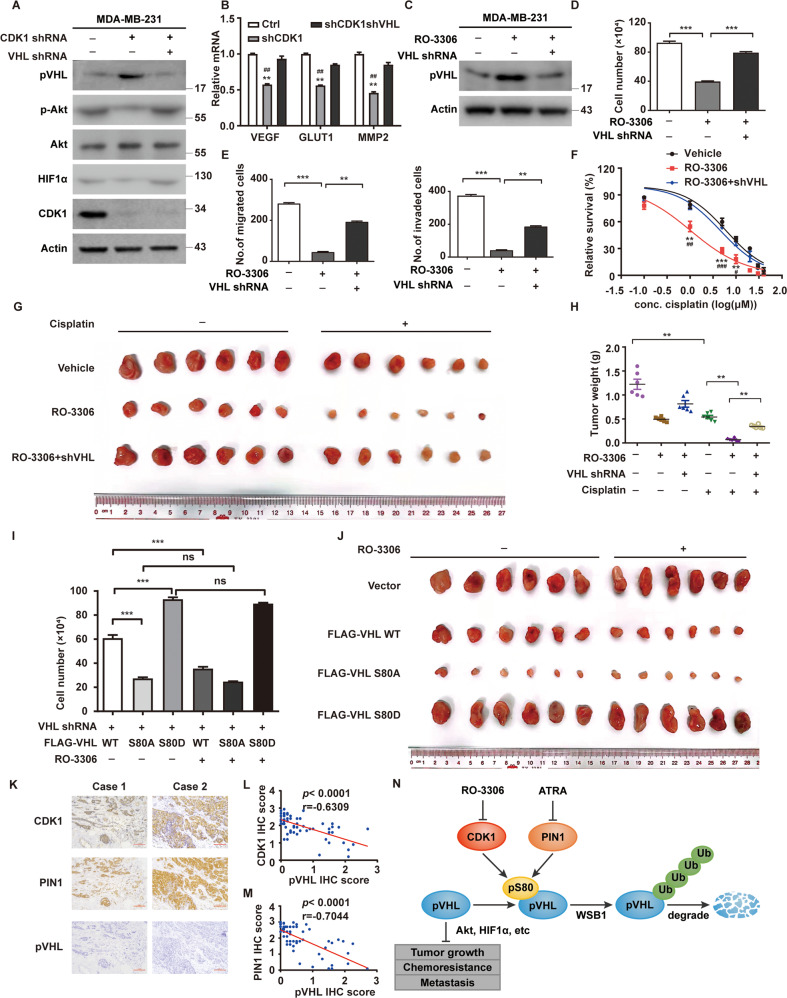
CDK1 promotes tumor progression through destabilizing pVHL and inversely correlates with pVHL expression in TNBC. **A** MDA-MB-231 cells stably expressing control, CDK1 or VHL shRNAs were generated and western blotting was performed with indicated antibodies. **B** Total RNA was isolated from MDA-MB-231 cells. Relative expression of VEGF, GLUT1 and MMP2 in cells stably expressing control, CDK1 or VHL shRNAs were determined by quantitative PCR. Results represent the mean ± SD of three independent experiments. **p < 0.01, shCDK1 vs Ctrl. ^##^*p* < 0.01, shCDK1shVHL vs shCDK1. **C** MDA-MB-231 cells were treated with RO-3306 and western blotting was performed with indicated antibodies. (**D**) Cell proliferation assay was performed in MDA-MB-231 cells. Results represent the mean ± SD of three independent experiments. ***p < 0.001, RO-3306 vs Vehicle, RO-3306 + shVHL vs RO-3306. **E** Transwell assays were performed to measure effects of RO-3306 on migratory (left) and invasive (right) abilities of BT-549 cells. Results represent the mean ± SD of three independent experiments. **p < 0.01, ***p < 0.001, RO-3306 vs Vehicle, RO-3306 + shVHL vs RO-3306. **F** MDA-MB-231 cells were treated with RO-3306. Cell survival was determined. Results represent the mean ± SD of four independent experiments. **p < 0.01, ***p < 0.001, RO-3306 vs Vehicle. ^#^*p* < 0.05, ^##^*p* < 0.01, ^###^*p* < 0.001, RO-3306 + shVHL vs RO-3306. **G**, **H** Mice with subcutaneously established tumors from MDA-MB-231 cells stably expressing indicated shRNAs were treated with Vehicle, RO-3306 (4 mg/kg), cisplatin (2 mg/kg) or combination. Mice were sacrificed to dissect xenograft tumors and measure tumor weights (**H**). Results represent the mean ± SD of six independent experiments. **p < 0.01, Vehicle + Cisplatin vs Vehicle, RO-3306 + Cisplatin vs Vehicle + Cisplatin, RO-3306 + shVHL + Cisplatin vs RO-3306 + Cisplatin. **I** Cell proliferation assay was performed in MDA-MB-231 cells. Results represent the mean ± SD of three independent experiments. ***p < 0.001, FLAG-VHL WT + RO-3306 vs FLAG-VHL WT Vehicle + , FLAG-VHL SA + RO-3306 vs FLAG-VHL SA + Vehicle, FLAG-VHL SD + RO-3306 vs FLAG-VHL SD + Vehicle, FLAG-VHL SA + Vehicle vs FLAG-VHL WT + Vehicle, FLAG-VHL SD + Vehicle vs FLAG-VHL WT + Vehicle. **J** Mice with subcutaneously established tumors from MDA-MB-231 cells stably expressing indicated plasmid were treated with Vehicle or RO-3306 (4 mg/kg). **K** Representative images of IHC staining of CDK1, PIN1 and pVHL in TNBC patient samples (*n* = 71). Scale bars, 100 μm. **L**, **M** Correlation analysis of CDK1 and pVHL expression levels as well as PIN1 and pVHL expression. **N** The working model to illustrate that CDK1 and PIN1 destabilize pVHL and promote tumor progression.

We next investigate whether CDK1 contributes to malignant progression in TNBC by destabilizing pVHL. As showed in Fig. 7A and Supplementary Fig. 8A, the depletion of CDK1 in TNBC cells significantly increased pVHL level and subsequently decreased the protein level of HIF1α and Akt phosphorylation, which could be markedly restored by the knockdown of *VHL*. In addition, the depletion of CDK1 decreased mRNA levels of HIF1α target genes *VEGF*, *GLUT1* and *MMP2* in a pVHL dependent manner (Fig. 7B and Supplementary Fig. 8B). Consistently, the treatment of RO-3306 in TNBC cells markedly reduced cell proliferation, migration and invasion ability (Fig. 7C–E and Supplementary Fig. 8C–F), along with increased cellular sensitivity to cisplatin and paclitaxel in vitro (Fig. 7F and Supplementary Fig. 8G–I), while the knockdown of *VHL* blocked such effects of RO-3306. Furthermore, results from xenograft experiments showed that RO-3306 inhibited tumor growth and increased sensitivity to cisplatin, which was also largely abrogated by the deficiency of *VHL* (Fig. 7G, H and Supplementary Fig. 8J).

We also found that the reconstitution of the S80A mutant in endogenous *VHL-*deficient cells displayed the strongest inhibitory effect on cell proliferation (Fig. 7I). *VHL* WT could inhibit cell proliferation to a lesser extent, which was remarkably enhanced by the treatment of RO-3306. However, the inhibition of CDK1 by RO-3306 did not cause any significant change in cells reconstituted with the *VHL* S80A or S80D mutant. Similar results were observed in xenograft animal experiments (Fig. 7J and Supplementary Fig. 8K, L). Together, our results uncover a previously unrecognized function of CDK1 to promote tumor progression through destabilizing pVHL in cancer harboring wild-type *VHL*.

### The overexpression of CDK1 and PIN1 are inversely correlated pVHL in TNBC specimens

Our results demonstrated that PIN1 and CDK1 cooperatively destabilize pVHL, thereby promoting tumor progression of TNBC. To further investigate the clinical relevance of PIN1/CDK1-pVHL axis in human TNBC tissues, we performed immunohistochemistry of an array of 71 human TNBC specimens. Notably, high expression of PIN1 (62/71, 87.32%) and CDK1 (60/71, 84.51%) were detected in most tumor specimens, while weak or none staining signals of pVHL was detected (55/71, 77.46%) (Fig. 7K). Importantly, the expression of CDK1 negatively correlated with pVHL protein expression in TNBC samples (Fig. 7L). Statistical significance and inverse correlation between PIN1 and pVHL was also observed (Fig. 7M). Moreover, the expression of PIN1 also negatively correlated with pVHL in ER^+^ breast cancer samples (Supplementary Fig. 8M, N). Collectively, our study demonstrates the novel mechanism of PIN1/CDK1 to cooperatively destabilize pVHL and promote tumor progression, thereby targeting PIN1 and CDK1 might be potential therapeutic strategies in the treatment of TNBC and other cancers with wild-type *VHL* (Fig. 7N).

## Discussion

Here, we reveal several unexpected findings with important clinical implications. We demonstrate the previously uncharacterized tumor-promoting activity of the CDK1/PIN1 axis through destabilizing pVHL in multiple cancer types harboring wild-type *VHL*. We discover that CDK1 catalyzed phosphorylation is a critical post-translational control of pVHL stability. We further provide preclinical evidence demonstrating that targeting CDK1/PIN1 axis is a common and effective approach to suppress tumor progression of TNBC and other cancers harboring wild-type *VHL* including TNBC (Fig. 7N).

Although previous reports showed that mutations in *VHL* are quite rare in lung cancer and hepatocellular carcinoma [12, 13], the status and function of *VHL* in breast cancer is much less studied. Our study discovered the *VHL* gene is largely wild-type in breast cancer and validates the therapeutic benefits of pVHL in TNBC, the most lethal subtypes of human breast cancers with limited therapeutic options. Intriguingly, we found that high expression of *VHL* is positively correlated with higher recurrence-free survival not only in TNBC, but also in other wild-type *VHL* harboring cancers, like pancreatic ductal adenocarcinoma and rectum adenocarcinoma, which is consistent with previous studies [23, 24]. Considering the well-established tumor-suppressive function of pVHL, to target pVHL stability might be an appealing strategy in the treatment of cancers harboring wild-type *VHL*. However, the development of pharmacological agents to directly stabilize pVHL in cancers is not yet successful.

The proline-directed phosphorylation (phosphor-Ser/Thr-Pro) is one common and central signaling mechanism in oncogenic pathways [37]. To date, PIN1 is the only known isomerase that specifically binds the phosphor-Ser/Thr-Pro motif of substrates and induces conformational changes, thereby regulating protein turnover, catalytic activity, dephosphorylation, protein–protein interactions and/or subcellular location of substrates [26, 38]. Here, our study reveals the previously unrecognized function of PIN1 in destabilizing pVHL and demonstrates the prospective clinical implication of directly targeting PIN1 by its inhibitor all-trans retinoic acid (ATRA) [28] in cancers with wild-type *VHL* including TNBC, other subtypes of breast cancers and pancreatic cancer, colorectal cancer, ovarian cancer and melanoma. The frequent aberrant upregulation and hyper-activation of PIN1 has been well documented in multiple cancers, indicating that PIN1 could be a common regulator to destabilize pVHL and attenuate its tumor-suppressive function, thus promoting the initiation and progression of cancers with wild-type *VHL* [31, 37, 39–41]. Thus, our study might expand the clinical implication of the pharmacological inhibition of PIN1 by ATRA in the treatment of cancers with wild-type *VHL*. Although our study is mainly focused in TNBC, further investigation need warranted especially in pancreatic cancer and other lethal cancers with worse prognosis and no targeted therapies.

In our study, we notice that the simultaneous depletion of *VHL* could markedly but not completely attenuate therapeutic benefits of the genetic ablation or the pharmacological inhibition of PIN1. We thus cannot rule out the possibility that additional mechanism is engaged in targeting PIN1 regulated tumor progression and metastasis. PIN1 has been reported to regulate tumorigenesis and tumor progression by stabilizing several oncogenic proteins such as p65 [42] and β-catenin [43]. PIN1 could also bind a variety of metabolic regulators including AMP-activated protein kinase, acetyl CoA carboxylase and pyruvate kinase 2 and regulate lipid/glucose metabolism in cancer cells, thereby to promote tumor progression [44]. A recent study showed that PIN1 stabilizes BRCA1 by preventing ubiquitination of Lys1037 of BRCA1. And the inhibition of PIN1 by All-trans retinoic acid destabilizes BRCA1 and extends benefit of PARP inhibitors to patients with homologous recombination-proficient tumors [21]. PIN1 also enhances STAT3-mediated EMT induced by Oncostatin M in breast cancer cells [45]. In addition, PIN1 may execute the oncogenic role through promoting the degradation or inactivation of tumor suppressors such as PML and FBXW7 [46]. Thus, the potential involvements of these substrates of PIN1 besides pVHL in metastasis and chemo-resistance of TNBC and other malignant caners need to be further investigated.

PIN1 specifically binds to the phosphor-S/TP motif of protein substrates. Here, we reveal a previously uncharacterized function of CDK1 as the direct kinase of pVHL. This CDK1-mediated phosphorylation of pVHL at Ser 80 is essential for its interaction with PIN1. CDK1 is originally identified as one important cyclin-dependent kinase (CDK) to regulate cell cycle progression in the majority of mammalian cells [47]. Emerging studies demonstrate that CDK1 regulates autophagy, protein synthesis [48, 49], epigenetic landscape [50], DNA damage response [51] and other cell cycle independent cellular processes [52]. It was reported that the high expression of CDK1 was significantly associated with a poor prognosis in breast cancer [53], which is consistent with our findings. In addition, the aberrant upregulation of CDK1 is also detected in lung cancer [54] and colorectal cancer [55] and positively correlated with pathological stage and lymphatic metastasis, although its exact substrates and the detailed mechanism remain not fully understood. CDK1-mediated mitotic phosphorylation of PBK was reported to be involved in cytokinesis and tumorigenesis [56], while one study indicated that CDK1 was identified to be a MYC synthetic-lethal gene and the inhibition of CDK1 selectively induces apoptosis in MYC-dependent cancer cell lines by upregulating the pro-apoptotic molecule BIM [57]. In addition, CDK1 could directly phosphorylate BRCA1 for the efficient formation of BRCA1 foci and thereby regulate BRCA1-mediated S phase checkpoint activation and HR. Thus, the inhibition of CDK1 could improve the response of BRCA-proficient breast cancer cells to PARP inhibition [58]. Here, we discover a novel cell cycle independent function of CDK1 to promote TNBC progression through promoting the interaction between pVHL and its E3 ligase WSB1, thereby targeting pVHL to ubiquitination and degradation. More intriguingly, the stabilization of pVHL by targeting CDK1 could also be a common regulatory mechanism in various types of cancer cells harboring wild-type *VHL*, including TNBC, pancreatic cancer, colorectal cancer and ovarian cancer (Fig. 6K, L).

Overall, our study provides important preclinical evidence that targeting PIN1/CDK1 axis is an appealing strategy to suppress tumor growth and metastasis, and sensitize cancer cells to chemotherapies by stabilizing pVHL in TNBC. More importantly, the cooperative regulation of pVHL stabilization by PIN1 and CDK1 might be a common regulatory mechanism in caners harboring wild-type *VHL*, since PIN1/CDK1-pVHL signaling axis exists not only in TNBC, but also in other cancer types harboring wild-type *VHL* including pancreatic cancer, colorectal cancer, ovarian cancer and other subtypes of human breast cancers. Given that, the pharmacological inhibition of PIN1 and CDK1 could be promisingly used in the therapy of these cancers especially TNBC and pancreatic cancers featured with worse prognosis and no targeted therapies. Further preclinical studies including PDX in vivo animal models and clinical studies are warranted to explore the therapeutic benefits of targeting the PIN1/CDK1-pVHL axis in the treatment of multiple human cancers with wild-type *VHL*.

## Supplementary information

SUPPLEMENTAL MATERIAL

SUPPLEMENTAL MATERIAL- ORIGINAL DATA

Reproducibility checklist

## Data Availability

All data generated or analyzed during this study are available within the article and Supplementary Files, or available from the authors upon request.
