# Supplementary material for: PIN1 and CDK1 cooperatively govern pVHL stability and suppressive functions
Source: Cell Death Differ. 2023 Feb 23;30(4):1082–95. doi: 10.1038/s41418-023-01128-x (PMC10070344; doi:10.1038/s41418-023-01128-x)
Supplement: Supplementary file 1 — SUPPLEMENTAL MATERIAL [file 41418_2023_1128_MOESM1_ESM.docx]

PIN1 and CDK1 Cooperatively Govern pVHL Stability and Suppressive Functions

Supplementary Text 1

**Supplementary Methods**

**Transwell assay:**

A transwell assay was used to determine migratory and invasive abilities of MDA-MB-231 and BT-549 cells. Transwell assays were carried out in 24-well plates using transwell chambers (8-μm pore size, Corning, NY, USA) precoated or not with Matrigel (dilute Matrigel in medium to 1 mg/mL). After being resuspended in serum-free medium, 4 × 10^4^ cells were seeded in the upper chamber, and 800 μL of medium containing 20% FBS was added to the lower transwell chamber. After culturing for 48 h, upper surfaces of transwells were wiped with cotton swabs. Cells in the transwell chamber were fixed and stained with crystal violet for 15 min, and then washed with PBS. Next, the number of migrating and invading cells was counted and imaged under a microscope (magnification: × 200; Olympus, Tokyo, Japan) in three random fields per well.

**Real-time quantitative PCR:**

Total RNA was isolated using TRIzol (Invitrogen), and 200 ng of RNA was reversed transcribed into cDNA using the FastKing gDNA Dispelling RT SuperMix (TIANGEN, Beijing, China). Real-time quantitative PCR was performed using the FastFire qPCR PreMix (SYBR Green) (TIANGEN). GAPDH was used as an internal control for normalization.

Primer sequences are listed:

GAPDH forward: GATCGAATTAAACCTTATCGTCGT,

reverse: GCAGCAGAACTTCCACTCGGT.

VHL forward: CTGCCCGTATGGCTCAACTT,

reverse: GTGTGTCCCTGCATCTCTGAAG.

MMP2 forward: TGACTTTCTTGGATCGGGTCG,

reverse: AAGCACCACATCAGATGACTG.

GLUT1 forward: CTTTGTGGCCTTCTTTGAAGT,

reverse: CCACACAGTTGCTCCACAT.

VEGF forward: GAGGAGCAGTTACGGTCTGTG,

reverse: TCCTTTCCTTAGCTGACACTTGT.

**Immunohistochemical staining:**

TNBC pathological tissue sections were obtained from the tissue bank from the First Affiliated Hospital of Jinan University (Guangdong, China) in accordance with the approval document of the Institutional Medical Ethics Committee (Ethics Approval License: JNUKY-2022-096). PIN1 (1:200), CDK1 (1:200) and VHL (1:100) staining were performed. Immunolabeling with a mixture of DAB solution, followed by counterstaining with hematoxylin. The immunostaining was randomly scored by two pathologists. The IHC score was calculated by combining the quantity score (percentage of positive stained tissues) with the staining intensity score. The staining intensity was scored as: 0 (negative), 1 (weak), 2 (moderate) and 3 (strong). The score for each tissue was calculated by multiplying the quantity with the intensity score. The χ2-test and the Pearson’s correlation coefficient were used for statistical analysis of the correlation between PIN1, CDK1 and pVHL.

Supplementary Fig. 1

pVHL suppresses tumor progression of TNBC


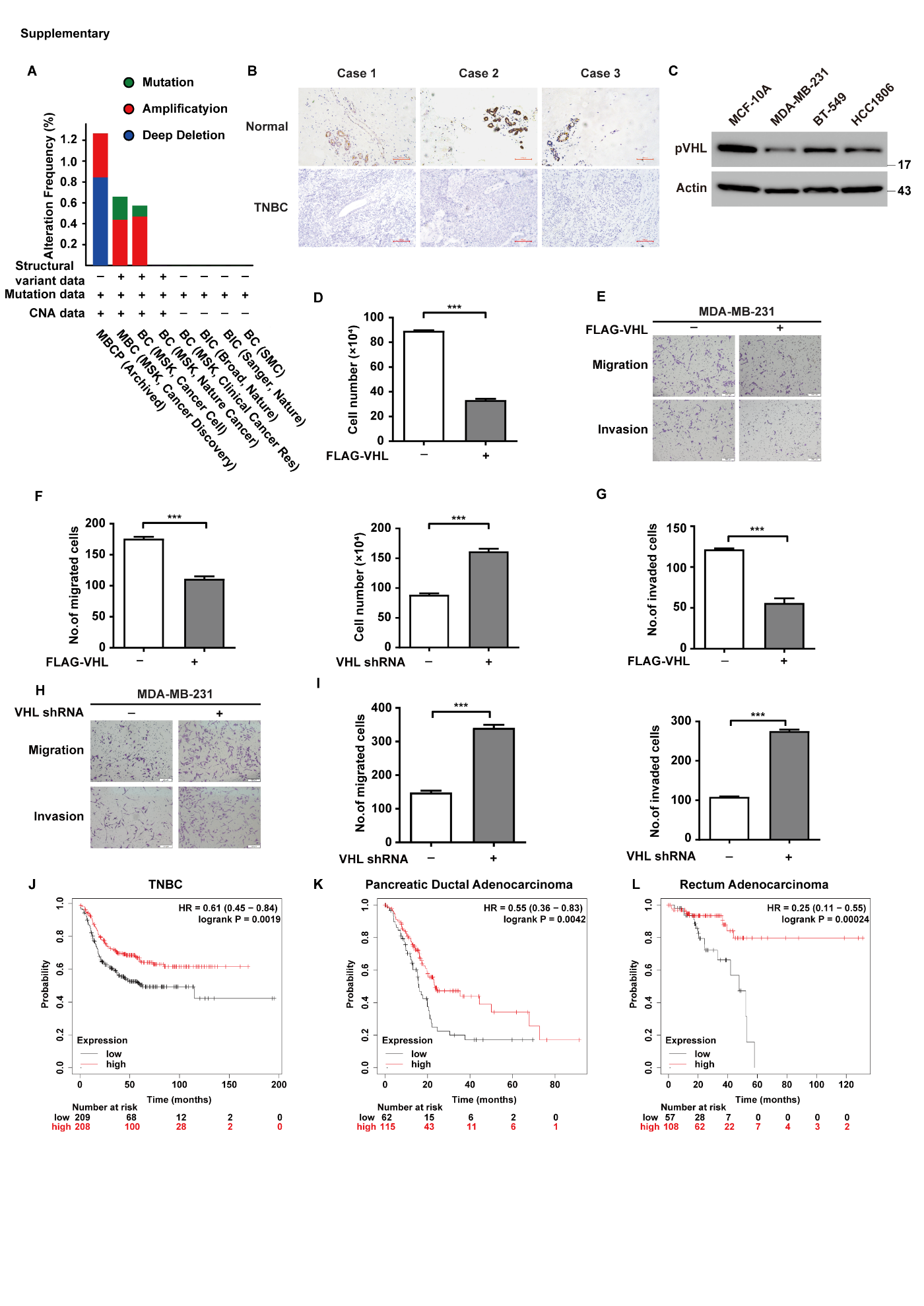


(**A**) Number of VHL mutations/copy number variations found in public database (cBioPortal database). MBCP represents The Metastatic Breast Cancer Project. MBC represents Metastatic Breast Cancer. BC represents Breast Cancer. BIC represents Breast Invasive Carcinoma. (**B**) Representative immunohistochemical images of pVHL in TNBC patient samples and normal breast tissues. Scale bars, 100 μm. (**C**) Cell lysates of several TNBC cell lines together with the MCF-10A (a human normal mammary epithelial cell) were collected and western blotting was performed with indicated antibodies. (**D**) Cell proliferation assay was performed in MDA-MB-231 cells. Results represent the mean ± SD of three independent experiments. ****p*<0.001, FLAG-VHL vs Vector. (**E, F**) Transwell assays were performed to measure effects of pVHL upregulation on migratory and invasive abilities of MDA-MB-231 cells. Results represent the mean ± SD of three independent experiments. ****p*<0.001, FLAG-VHL vs Vector. Scale bars, 200 μm. (**G**) Cell proliferation assay was performed in MDA-MB-231 cells. Results represent the mean ± SD of three independent experiments. ****p*<0.001, shVHL vs Ctrl. (**H, I**) Transwell assays were performed to measure effects of pVHL downregulation on migratory and invasive abilities of MDA-MB-231 cells. Results represent the mean ± SD of three independent experiments. ****p*<0.001, shVHL vs Ctrl. Scale bars, 200 μm. (**J-L**) Kaplan-Meier graph of TNBC, pancreatic ductal adenocarcinoma and rectum adenocarcinoma patients, stratified according to high or low expression levels of *VHL*.

Supplementary Fig. 2

PIN1 regulates the stability of pVHL by affecting its ubiquitination


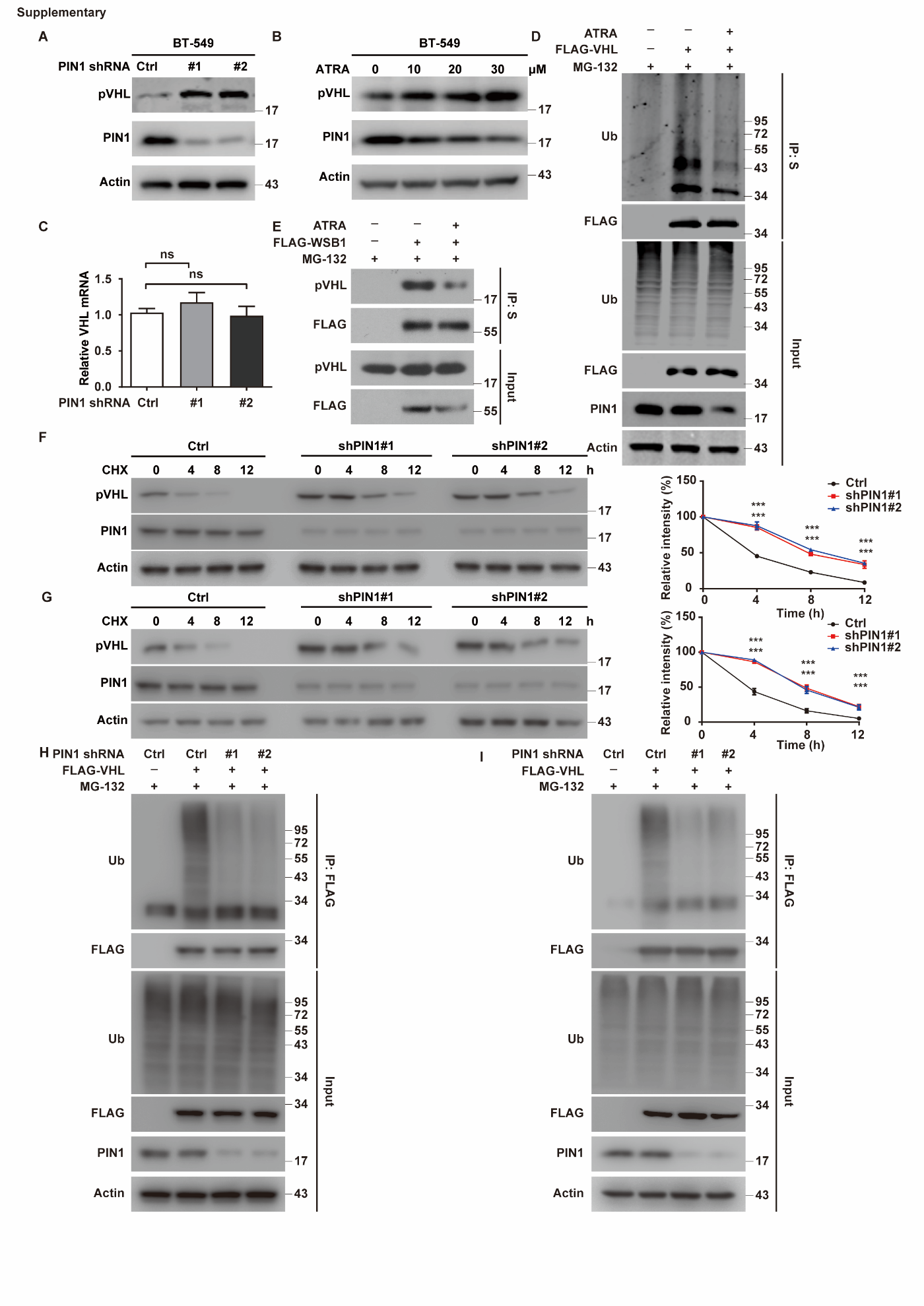


(**A**) BT-549 cells stably expressing control or PIN1 shRNAs were generated and western blotting was performed with indicated antibodies. (**B**) BT-549 cells were treated with ATRA and western blotting was performed with indicated antibodies. (**C**) Total RNA was isolated from cells in (A). Relative expression of VHL in cells stably expressing control or PIN1 shRNAs was determined by quantitative PCR. Results represent the mean ± SD of three independent experiments. shPIN1#1 vs Ctrl, shPIN1#2 vs Ctrl. (**D**) HEK293T cells were transfected with indicated constructs and treated with Vehicle or ATRA for 24 h in the presence of MG-132. Cell lysates were immunoprecipitated with S-protein agarose, and the polyubiquitylated pVHL was detected by anti-ubiquitin antibody. (**E**) Cells were transfected with indicated plasmids and treated with Vehicle or ATRA for 24 h, cell lysates were subjected to immunoprecipitation with S-protein agarose and western blotting was performed. (**F, G**) Cycloheximide pulse-chase assay was performed in MCF-7 (F) and SK-BR-3 (G) cells and results were quantified (right). Results represent the mean ± SD of three independent experiments. ****p*<0.001, shPIN1#1 vs Ctrl, shPIN1#2 vs Ctrl. (**H, I**) MCF-7 (H) and SK-BR-3 (I) cells stably expressing control or PIN1 shRNAs were generated and transfected with indicated plasmids and treated with MG-132 for 8 h before cell lysates were immunoprecipitated with anti-FLAG affinity gel, and the polyubiquitylated pVHL was detected by anti-ubiquitin antibody.

Supplementary Fig. 3

The depletion of PIN1 suppresses tumor progression of TNBC through pVHL

**
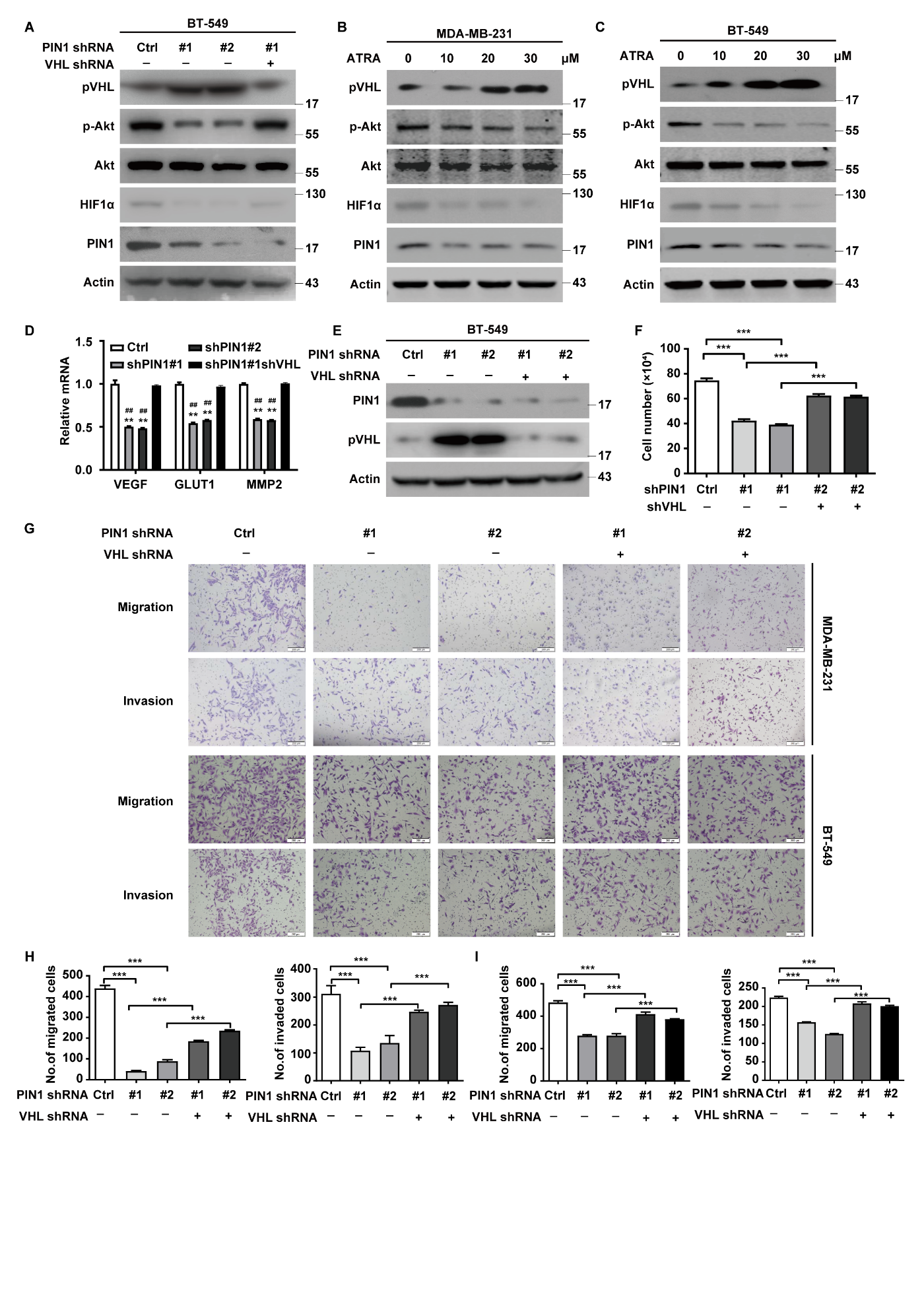
**

(**A**) BT-549 cells stably expressing control or PIN1 shRNAs were generated and western blotting was performed with indicated antibodies. (**B, C**) MDA-MB-231 or BT-549 cells were treated with ATRA and western blotting was performed with indicated antibodies. (**D**) Total RNA was isolated from BT-549 cells. Relative expression of VEGF, GLUT1 and MMP2 in cells stably expressing control, PIN1 or VHL shRNAs were determined by quantitative PCR. Results represent the mean ± SD of three independent experiments. ***p*<0.01, shPIN1#1 vs Ctrl, shPIN1#2 vs Ctrl. ##*p*<0.01, shPIN1#1shVHL vs shPIN1#1, shPIN1#1shVHL vs shPIN1#2. (**E**) Cells were infected with indicated shRNAs. Western blotting was performed. (**F**) Cell proliferation assay was performed in BT-549 cells. Results represent the mean ± SD of three independent experiments. ****p*<0.001, shPIN1#1 vs Ctrl, shPIN1#2 vs Ctrl, shPIN1#1shVHL vs shPIN1#1, shPIN1#1shVHL vs shPIN1#2. (**G-I**) Transwell assays were performed to measure effects of PIN1 downregulation on migratory and invasive abilities of MDA-MB-231 (H) or BT-549 (I) cells. Results represent the mean ± SD of three independent experiments. ****p*<0.001, shPIN1#1 vs Ctrl, shPIN1#2 vs Ctrl, shPIN1#1shVHL vs shPIN1#1, shPIN1#1shVHL vs shPIN1#2. Scale bars, 200 μm.

Supplementary Fig. 4

The pharmacological inhibition of PIN1 by ATRA suppresses tumor progression of TNBC through pVHL


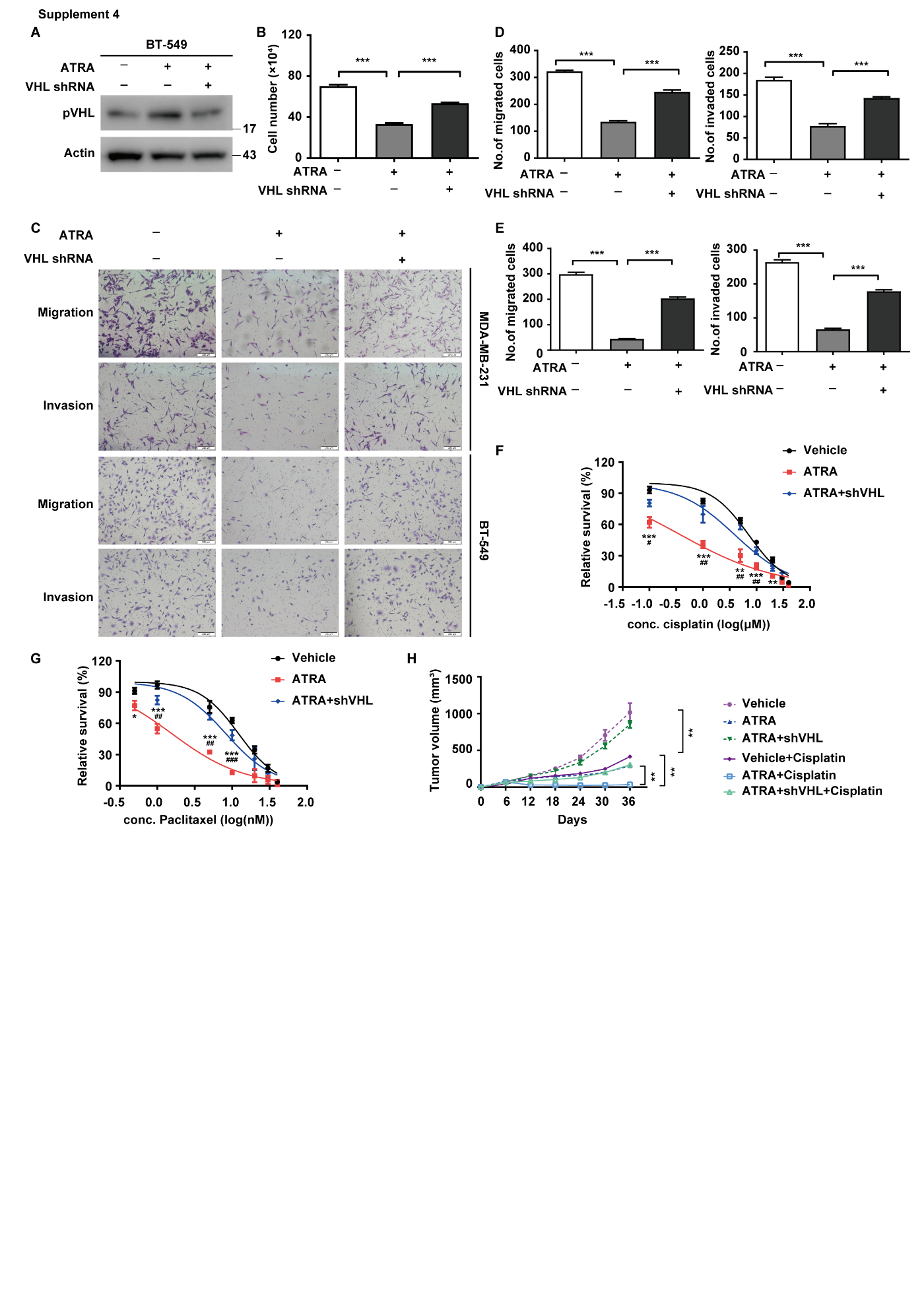


(**A**) BT-549 cells were treated with ATRA and western blotting was performed with indicated antibodies. (**B**) Cell proliferation assay was performed in BT-549 cells. Results represent the mean ± SD of three independent experiments. ****p*<0.001, ATRA vs Vehicle, ATRA + shVHL vs ATRA. (**C-E**) Transwell assays were performed to measure effects of ATRA on migratory and invasive abilities of MDA-MB-231 (D) or BT-549 (E) cells. Results represent the mean ± SD of three independent experiments. ****p*<0.001, ATRA vs Vehicle, ATRA + shVHL vs ATRA. Scale bars, 200 μm. (**F, G**) BT-549 cells were treated with ATRA. Cell survival was determined. Results represent the mean ± SD of four independent experiments. **p*<0.05, ***p*<0.01, ****p*<0.001, ATRA vs Vehicle. #*p*<0.05, ##*p*<0.01, ###*p*<0.001, ATRA + shVHL vs ATRA. (**H**) Primary tumor size was measured and quantified. Results represent the mean ± SD of six independent experiments. ***p*<0.01, Vehicle + Cisplatin vs Vehicle, ATRA + Cisplatin vs Vehicle + Cisplatin, ATRA + shVHL + Cisplatin vs ATRA + Cisplatin.

Supplementary Fig. 5

The phosphorylation of pVHL at Ser80 promotes the degradation of pVHL by ubiquitin-proteasome system

**
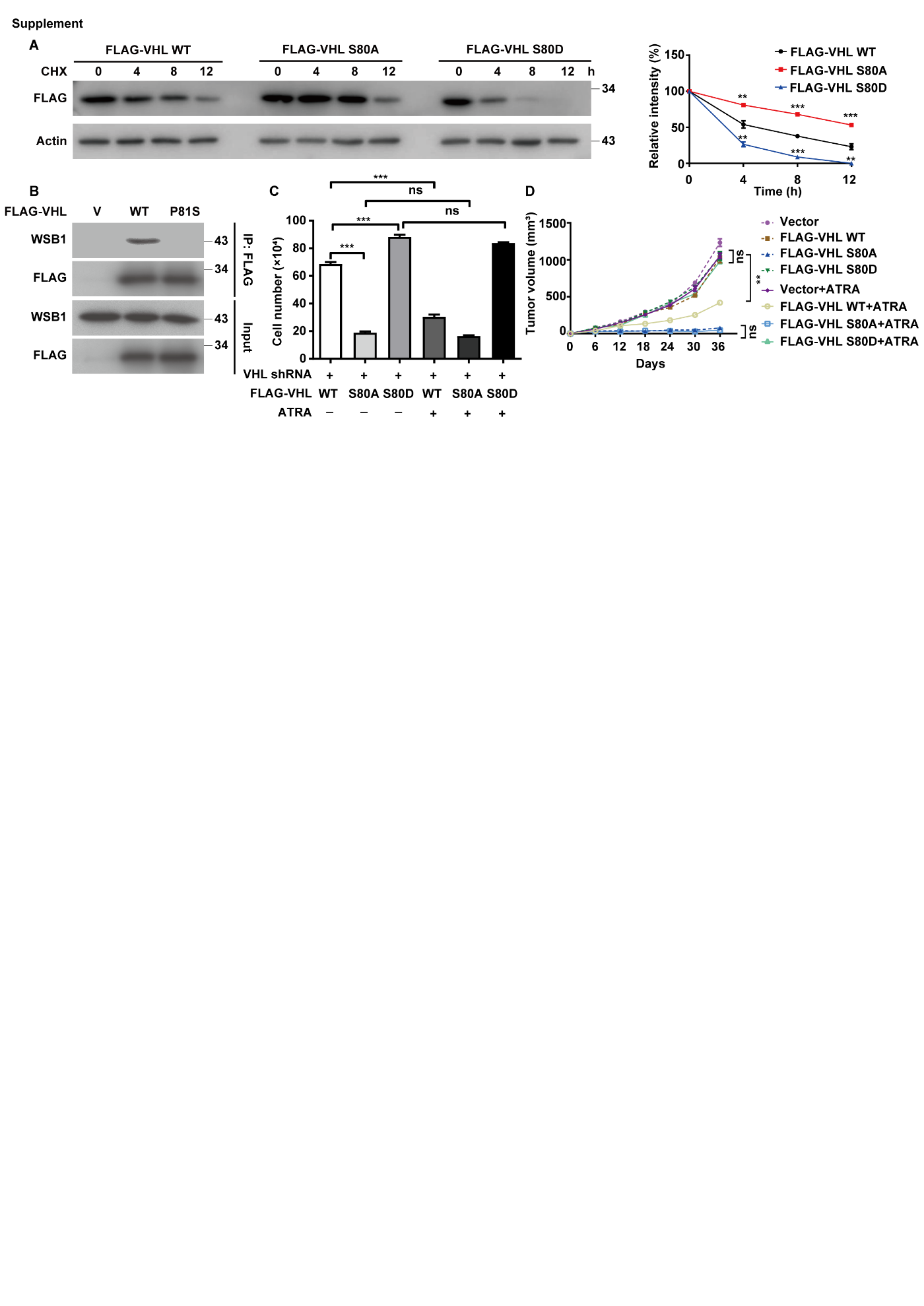
**

(**A**) Cycloheximide pulse-chase assay was performed in BT-549 cells and results were quantified (right). Results represent the mean ± SD of three independent experiments. ***p*<0.01, ****p*<0.001, FLAG-VHL SA vs FLAG-VHL WT, FLAG-VHL SD vs FLAG-VHL WT. (**B**) Cell lysates were subjected to immunoprecipitation with anti-FLAG affinity gel and western blotting was performed. (**C**) Cell proliferation assay was performed in MDA-MB-231 cells. Results represent the mean ± SD of three independent experiments. ****p*<0.001, FLAG-VHL WT + ATRA vs FLAG-VHL WT + Vehicle, FLAG-VHL SA + ATRA vs FLAG-VHL SA + Vehicle, FLAG-VHL SD + ATRA vs FLAG-VHL SD + Vehicle, FLAG-VHL SA + Vehicle vs FLAG-VHL WT + Vehicle, FLAG-VHL SD + Vehicle vs FLAG-VHL WT + Vehicle. (**D**) Primary tumor size was measured and quantified. Results represent the mean ± SD of six independent experiments. ***p*<0.01, WT + ATRA vs WT + Vehicle, SA + ATRA vs SA + Vehicle, SD + ATRA vs SD + Vehicle.

Supplementary Fig. 6

CDK1 directly binds and phosphorylates pVHL

**
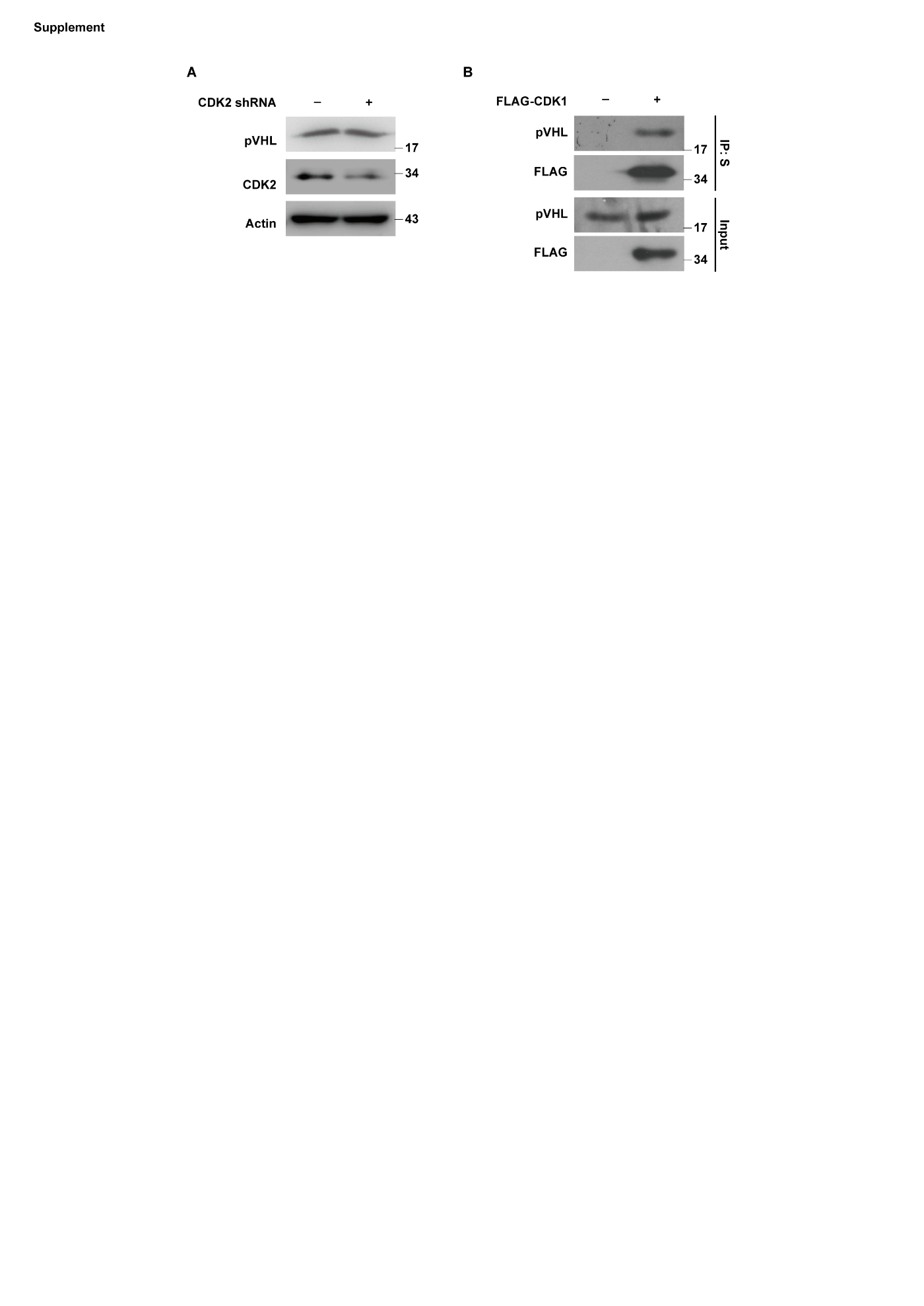
**

(**A**) MDA-MB-231 cells were infected with indicated shRNAs. Western blotting was performed. (**B**) Cells were transfected with indicated plasmids, cell lysates were subjected to immunoprecipitation with S-protein agarose and western blotting was performed.

Supplementary Fig. 7

CDK1 phosphorylates pVHL and promotes its degradation by ubiquitin-proteasome system


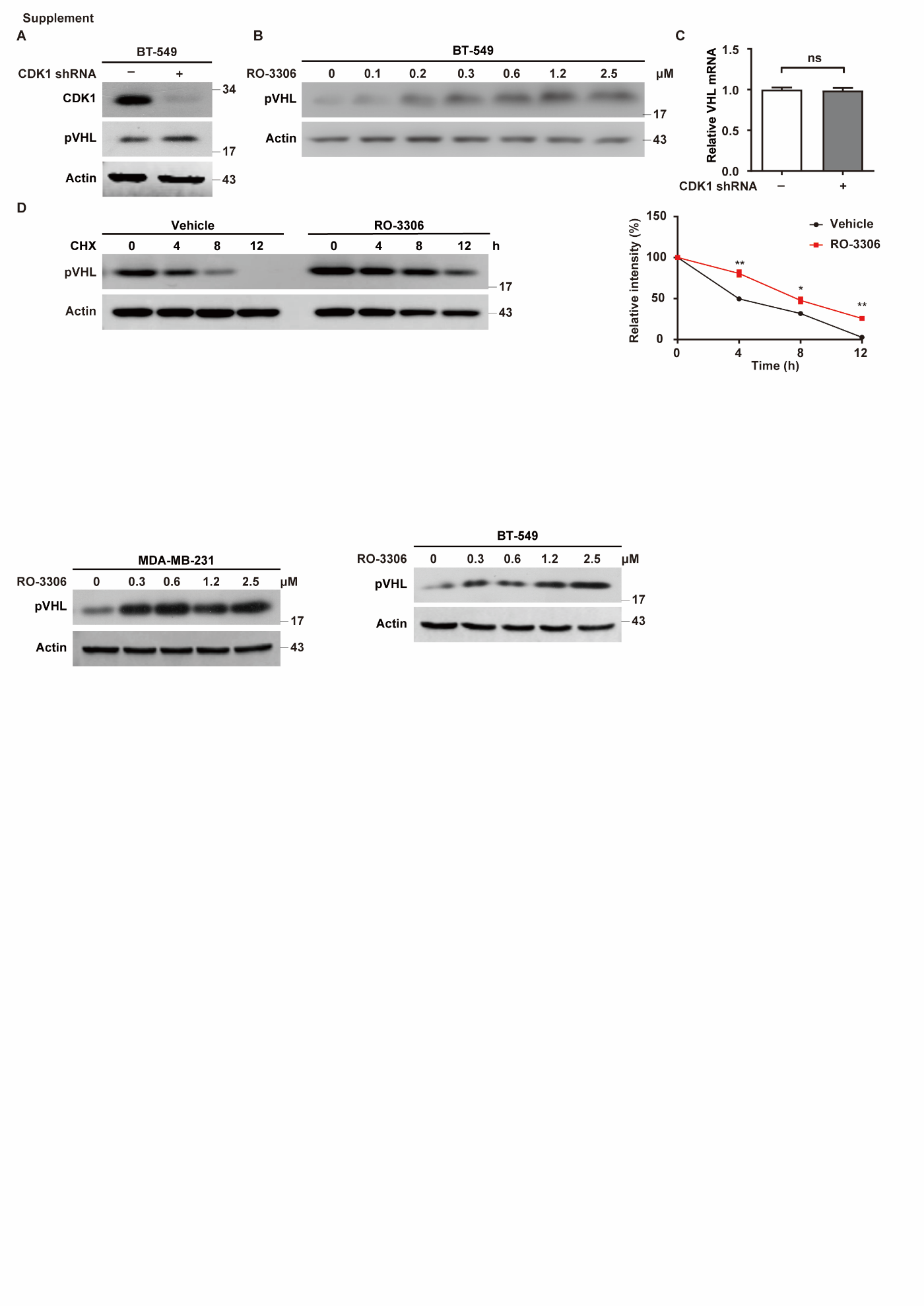


(**A**) BT-549 cells were infected with indicated shRNAs. Western blotting was performed. (**B**) BT-549 cells were treated with RO-3306 and western blotting was performed with indicated antibodies. (**C**) Total RNA was isolated from cells in (A). Relative expression of VHL in cells stably expressing control or CDK1 shRNAs was determined by quantitative PCR. Results represent the mean ± SD of three independent experiments. shCDK1 vs Ctrl. (**D**) BT-549 cells were treated with RO-3306 for 24 h. Cycloheximide pulse-chase assay was performed in cells and results were quantified (right). Results represent the mean ± SD of three independent experiments. **p*<0.05, ***p*<0.01, RO-3306 vs Vehicle.

Supplementary Fig. 8

CDK1 promotes tumor progression of TNBC through destabilizing pVHL

**
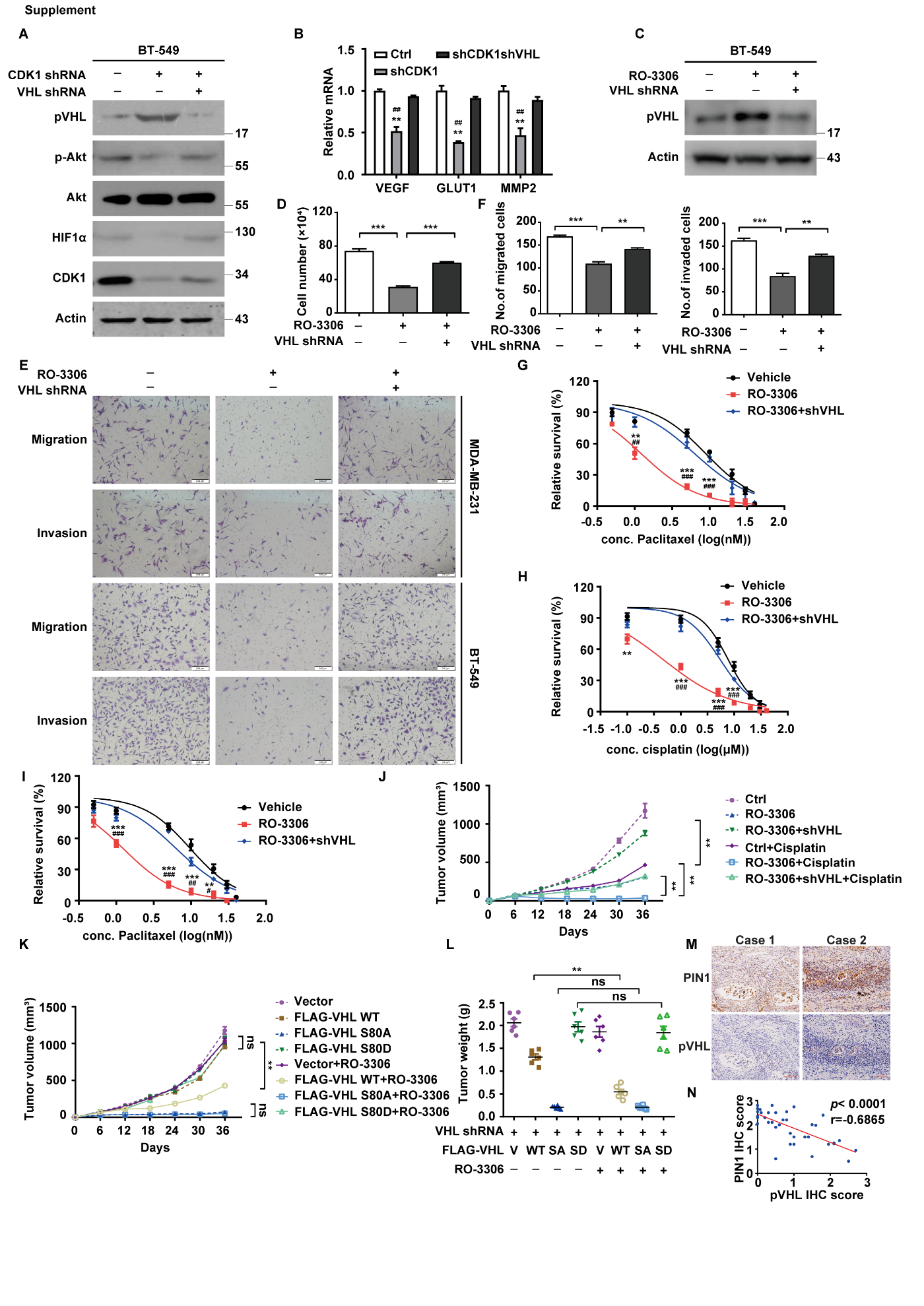
**

(**A**) BT-549 cells stably expressing control, CDK1 or VHL shRNAs were generated and western blotting was performed with indicated antibodies. (**B**) Total RNA was isolated from BT-549 cells. Relative expression of VEGF, GLUT1 and MMP2 in cells stably expressing control, CDK1 or VHL shRNAs were determined by quantitative PCR. Results represent the mean ± SD of three independent experiments. ***p*<0.01, shCDK1 vs Ctrl. ##*p*<0.01, shCDK1shVHL vs shCDK1. (**C**) BT-549 cells were treated with RO-3306 and western blotting was performed with indicated antibodies. (**D**) Cell proliferation assay was performed in BT-549 cells. Results represent the mean ± SD of three independent experiments. ****p*<0.001, RO-3306 vs Vehicle, RO-3306 + shVHL vs RO-3306. (**E, F**) Transwell assays were performed to measure effects of RO-3306 on migratory and invasive abilities of MDA-MB-231 (F) or BT-549 cells. Results represent the mean ± SD of three independent experiments. ****p*<0.001, RO-3306 vs Vehicle, RO-3306 + shVHL vs RO-3306. Scale bars, 200 μm. (**G**) MDA-MB-231 cells were treated with RO-3306. Cell survival was determined. Results represent the mean ± SD of four independent experiments. ***p*<0.01, ****p*<0.001, RO-3306 vs Vehicle. ##*p*<0.01, ###*p*<0.001, RO-3306 + shVHL vs RO-3306. (**H, I**) BT-549 cells were treated with RO-3306. Cell survival was determined. Results represent the mean ± SD of four independent experiments. ***p*<0.01, ****p*<0.001, RO-3306 vs Vehicle. #*p*<0.05, ##*p*<0.01, ###*p*<0.001, RO-3306 + shVHL vs RO-3306. (**J**) Primary tumor size was measured and quantified. Results represent the mean ± SD of six independent experiments. ***p*<0.01, Vehicle + Cisplatin vs Vehicle, RO-3306 + Cisplatin vs Vehicle + Cisplatin, RO-3306 + shVHL + Cisplatin vs RO-3306 + Cisplatin. (**K**) Primary tumor size was measured and quantified. Results represent the mean ± SD of six independent experiments. ***p*<0.01, WT + RO-3306 vs WT + Vehicle, SA + RO-3306 vs SA + Vehicle, SD + RO-3306 vs SD + Vehicle. (**L**) Mice were sacrificed to dissect xenograft tumors and measure tumor weights. Results represent the mean ± SD of six independent experiments. ***p*<0.01, WT + RO-3306 vs WT + Vehicle, SA + RO-3306 vs SA + Vehicle, SD + RO-3306 vs SD + Vehicle. (**M**) Representative images of IHC staining of PIN1 and pVHL in ER^+^ breast cancer patient samples (n=40). Scale bars, 100 μm. (**N**) Correlation analysis of PIN1 and pVHL expression levels.
