# Supplementary material for: PIN1 and CDK1 cooperatively govern pVHL stability and suppressive functions
Source: Cell Death Differ. 2023 Feb 23;30(4):1082–95. doi: 10.1038/s41418-023-01128-x (PMC10070344; doi:10.1038/s41418-023-01128-x)
Supplement: Supplementary file 2 — SUPPLEMENTAL MATERIAL- ORIGINAL DATA [file 41418_2023_1128_MOESM2_ESM.docx]

PIN1 and CDK1 Cooperatively Govern pVHL Stability and Suppressive Functions

Supplementary Text 2

Supplementary Fig. 9


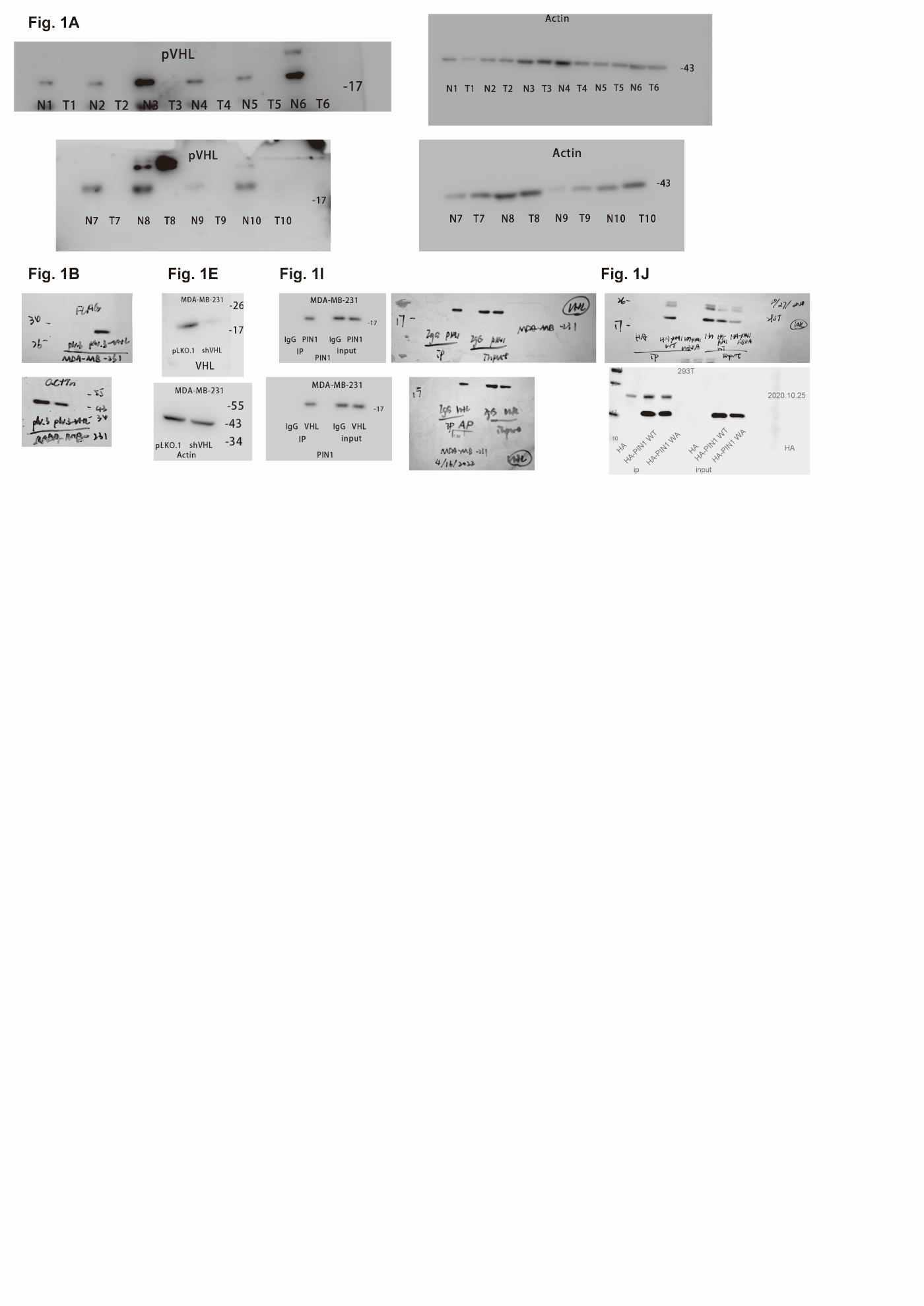


Supplementary Fig. 9: Original scan of the blots presented in the main text. Related to Fig. 1.

Supplementary Fig. 10


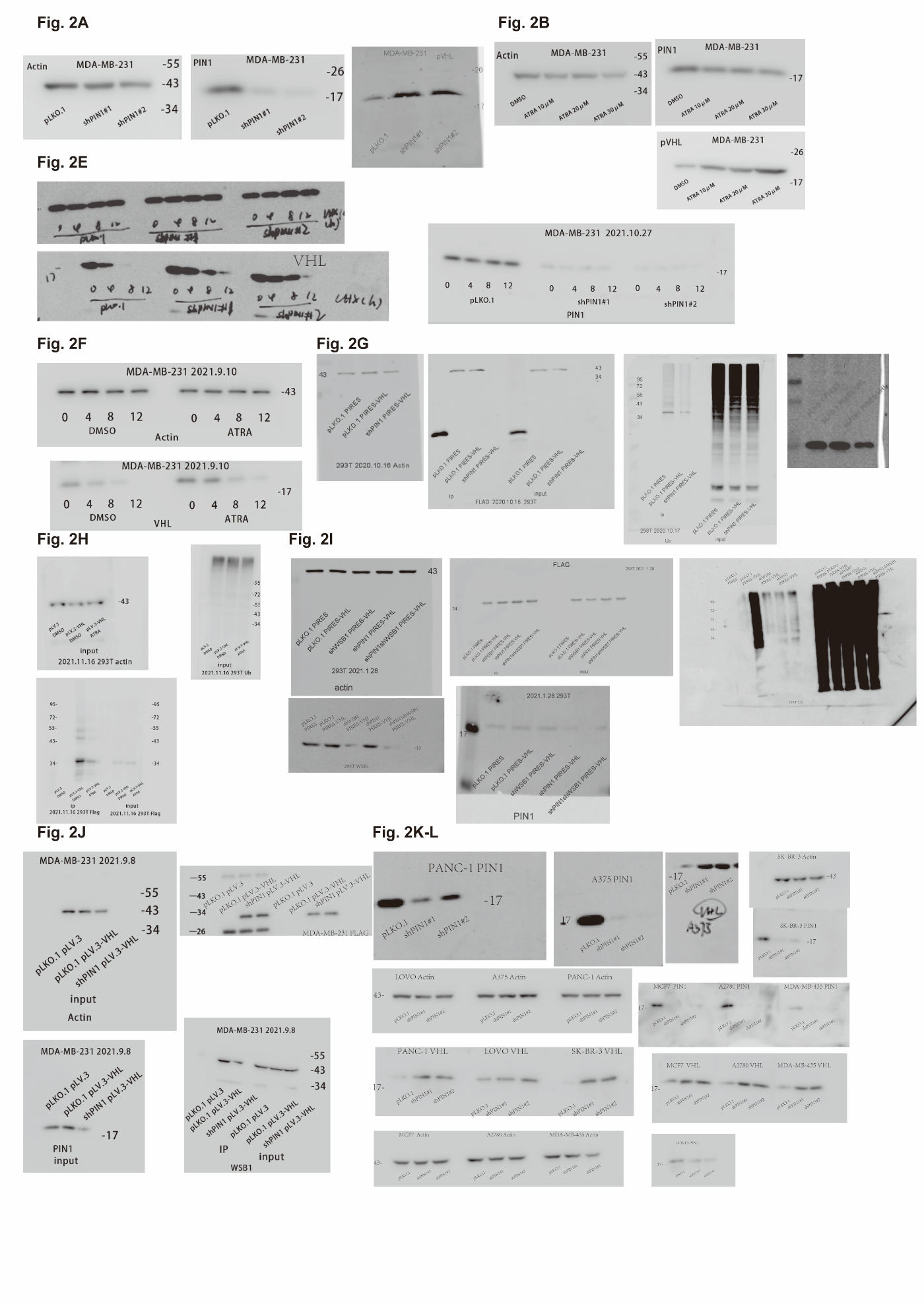


Supplementary Fig. 10: Original scan of the blots presented in the main text. Related to Fig. 2.

Supplementary Fig. 11


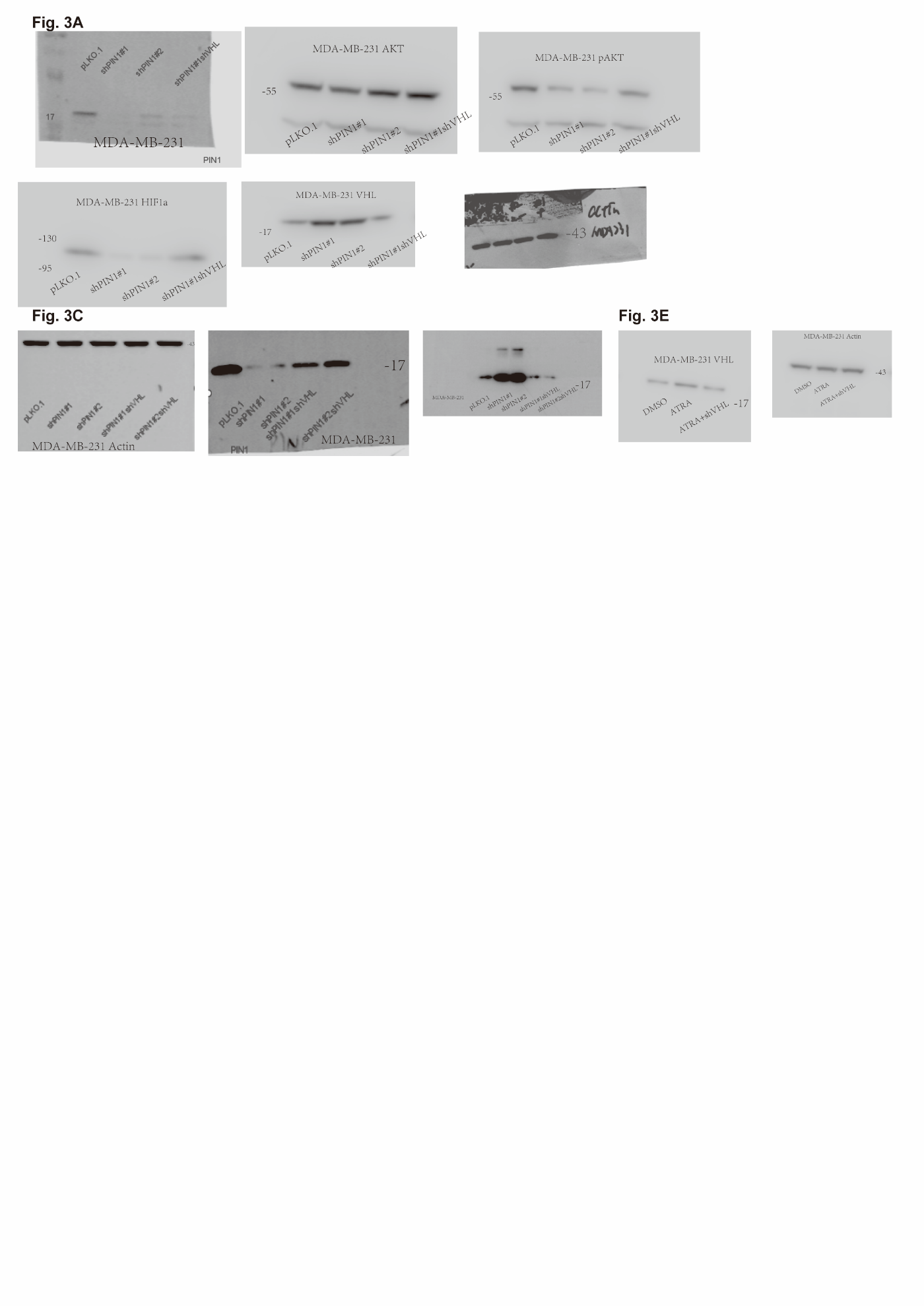


Supplementary Fig. 11: Original scan of the blots presented in the main text. Related to Fig. 3.

Supplementary Fig. 12


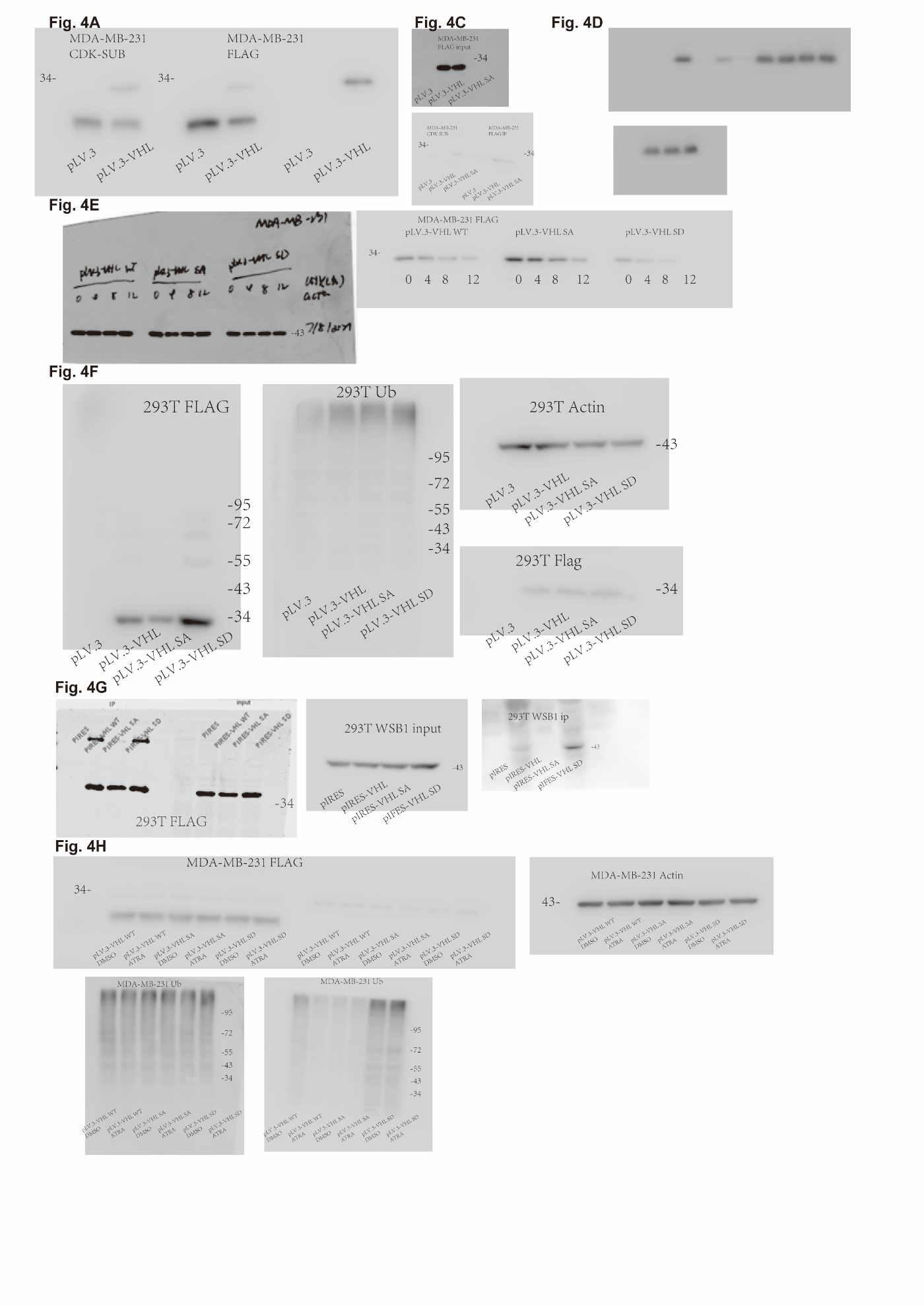


Supplementary Fig. 12: Original scan of the blots presented in the main text. Related to Fig. 4.

Supplementary Fig. 13


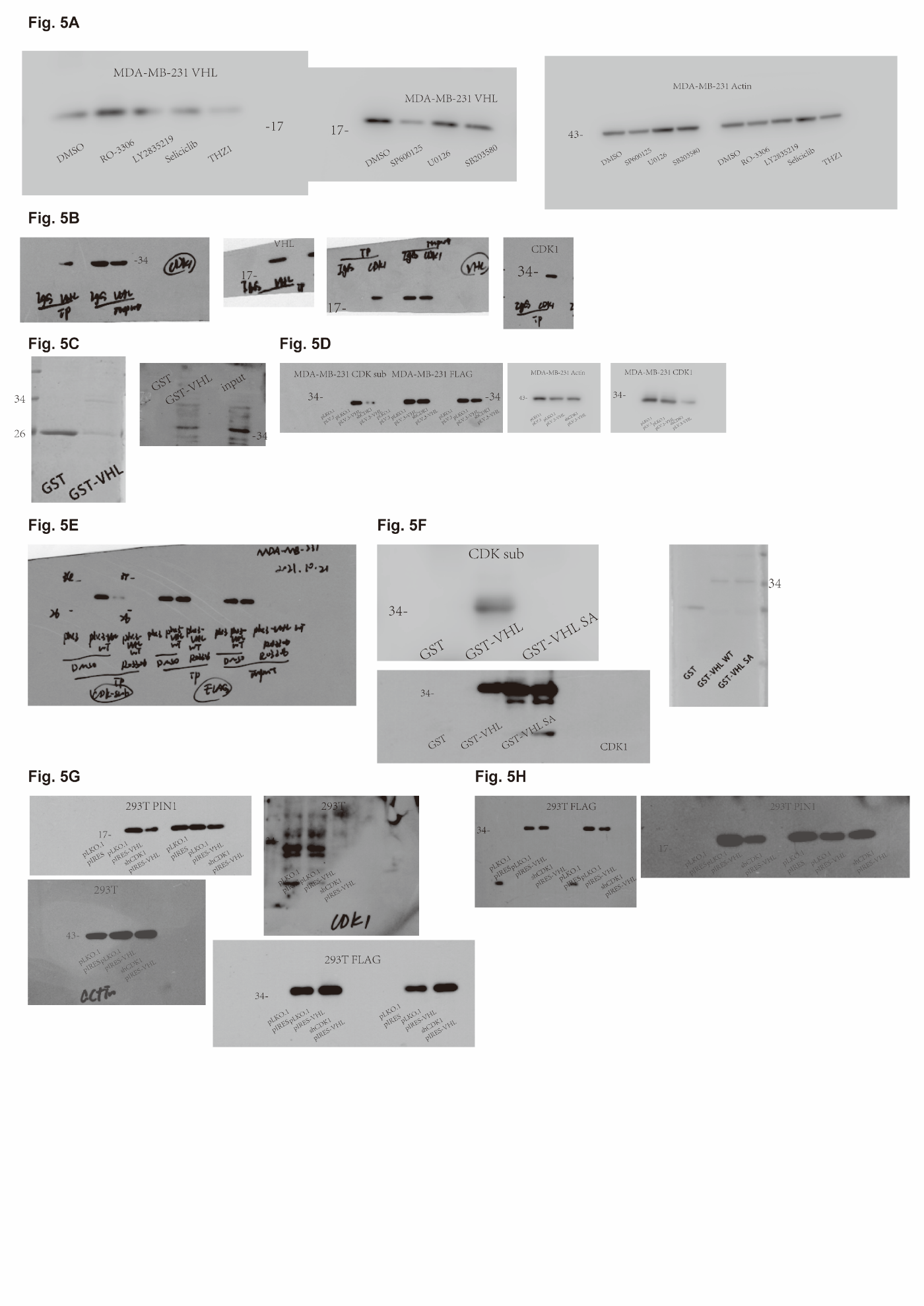


Supplementary Fig. 13: Original scan of the blots presented in the main text. Related to Fig. 5.

Supplementary Fig. 14


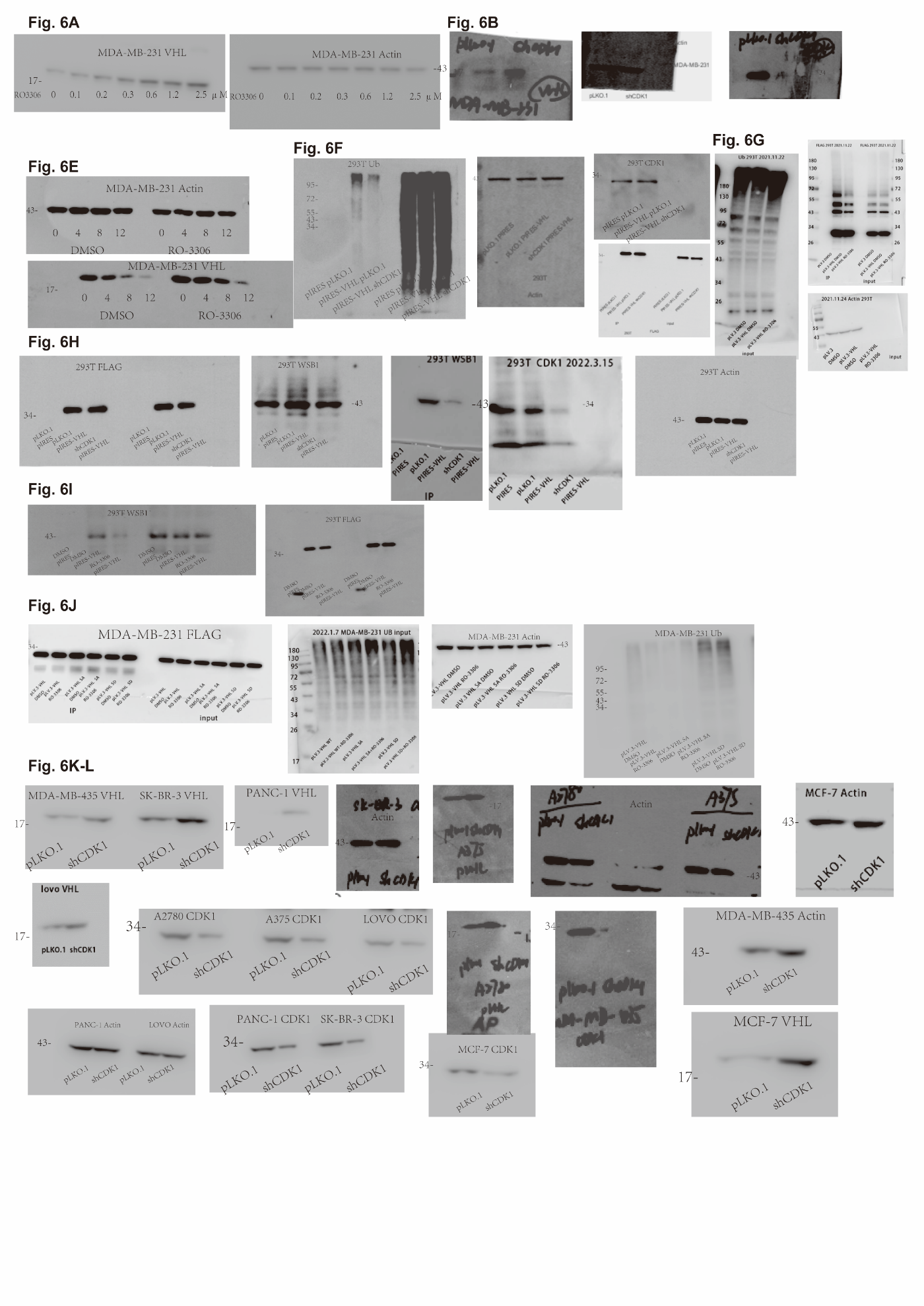


Supplementary Fig. 14: Original scan of the blots presented in the main text. Related to Fig. 6.

Supplementary Fig. 15


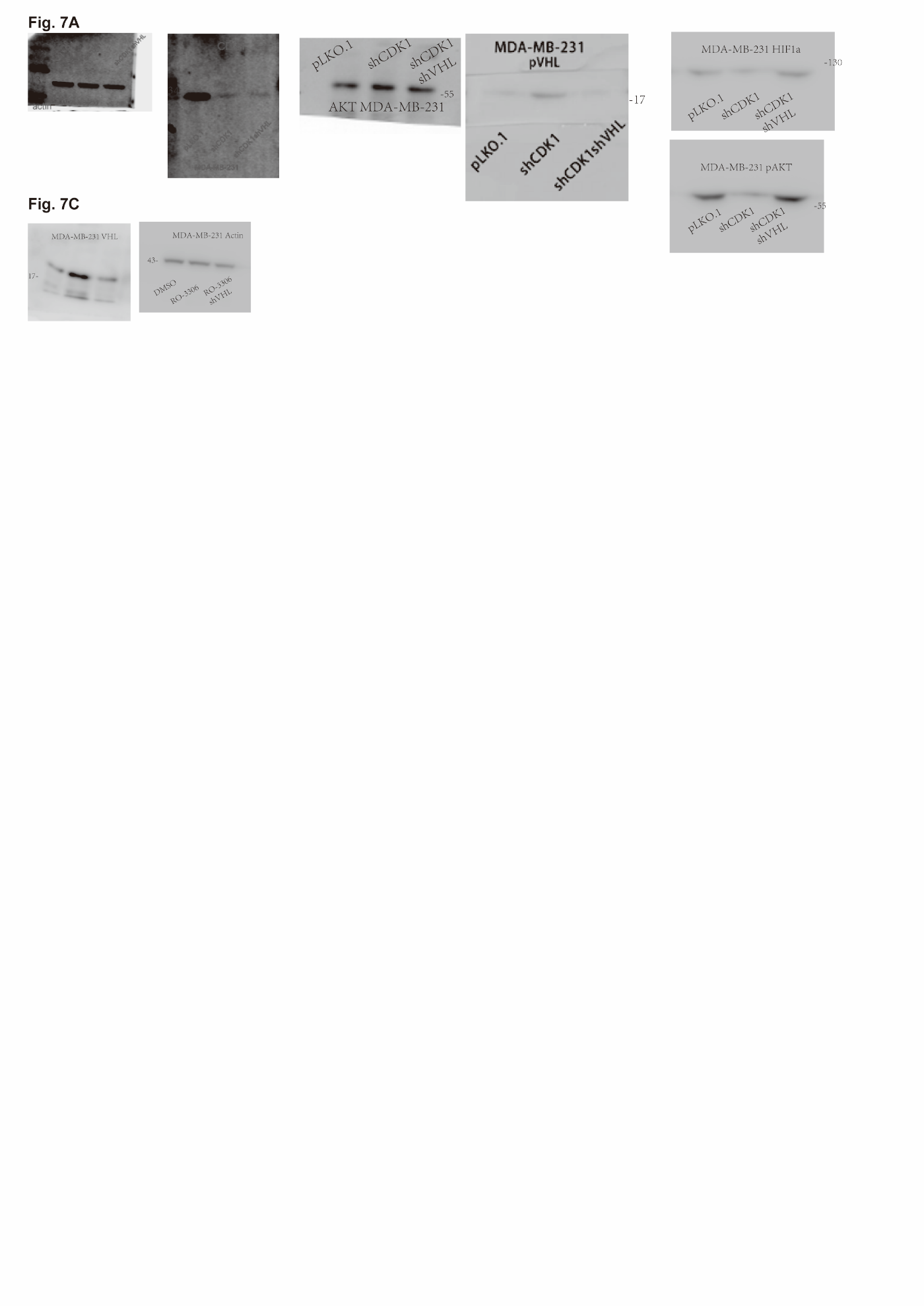


Supplementary Fig. 15: Original scan of the blots presented in the main text. Related to Fig. 7.

Supplementary Fig. 16


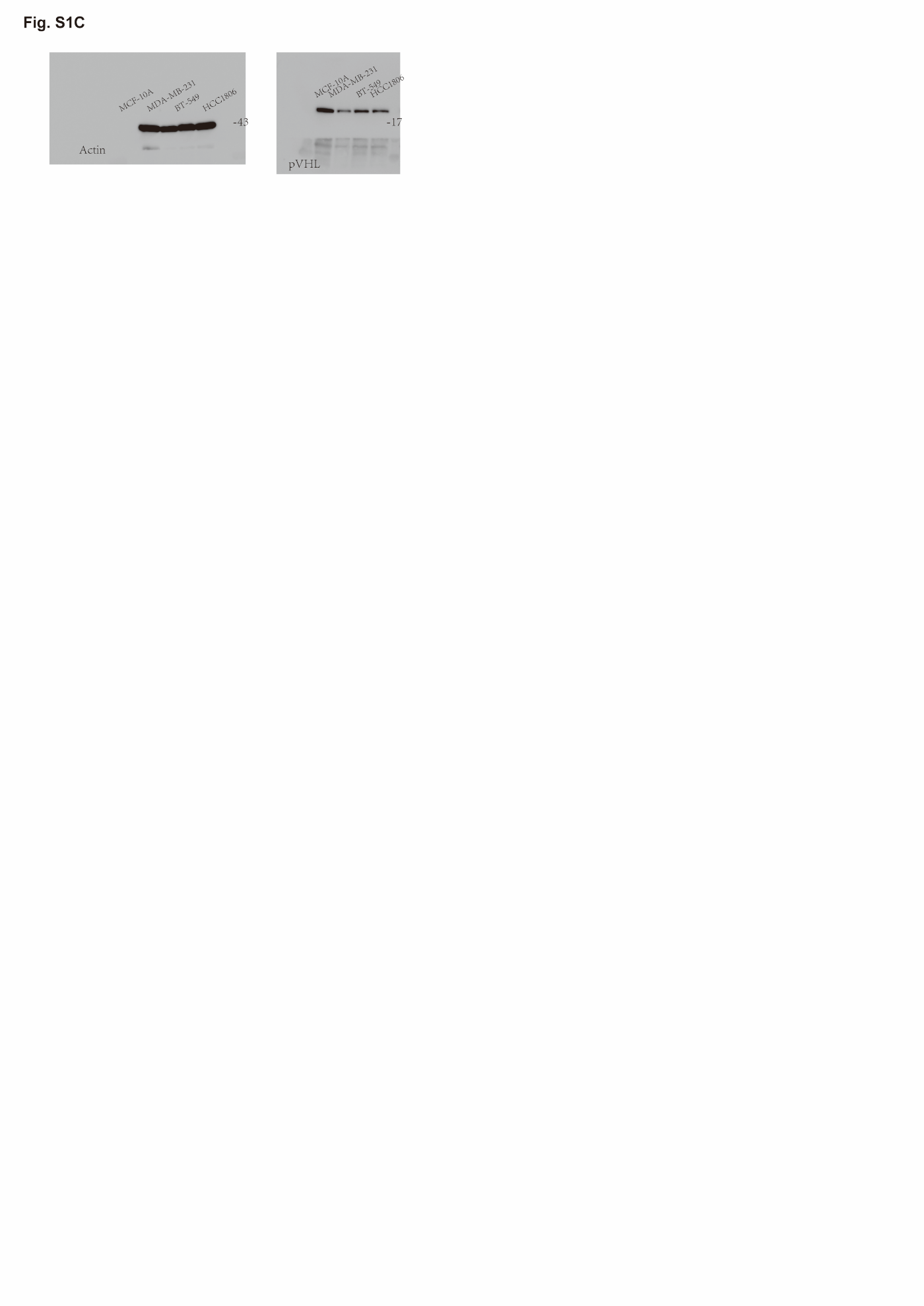


Supplementary Fig. 16: Original scan of the blots presented in the Supplementary Text 1. Related to Supplementary Fig. 1.

Supplementary Fig. 17


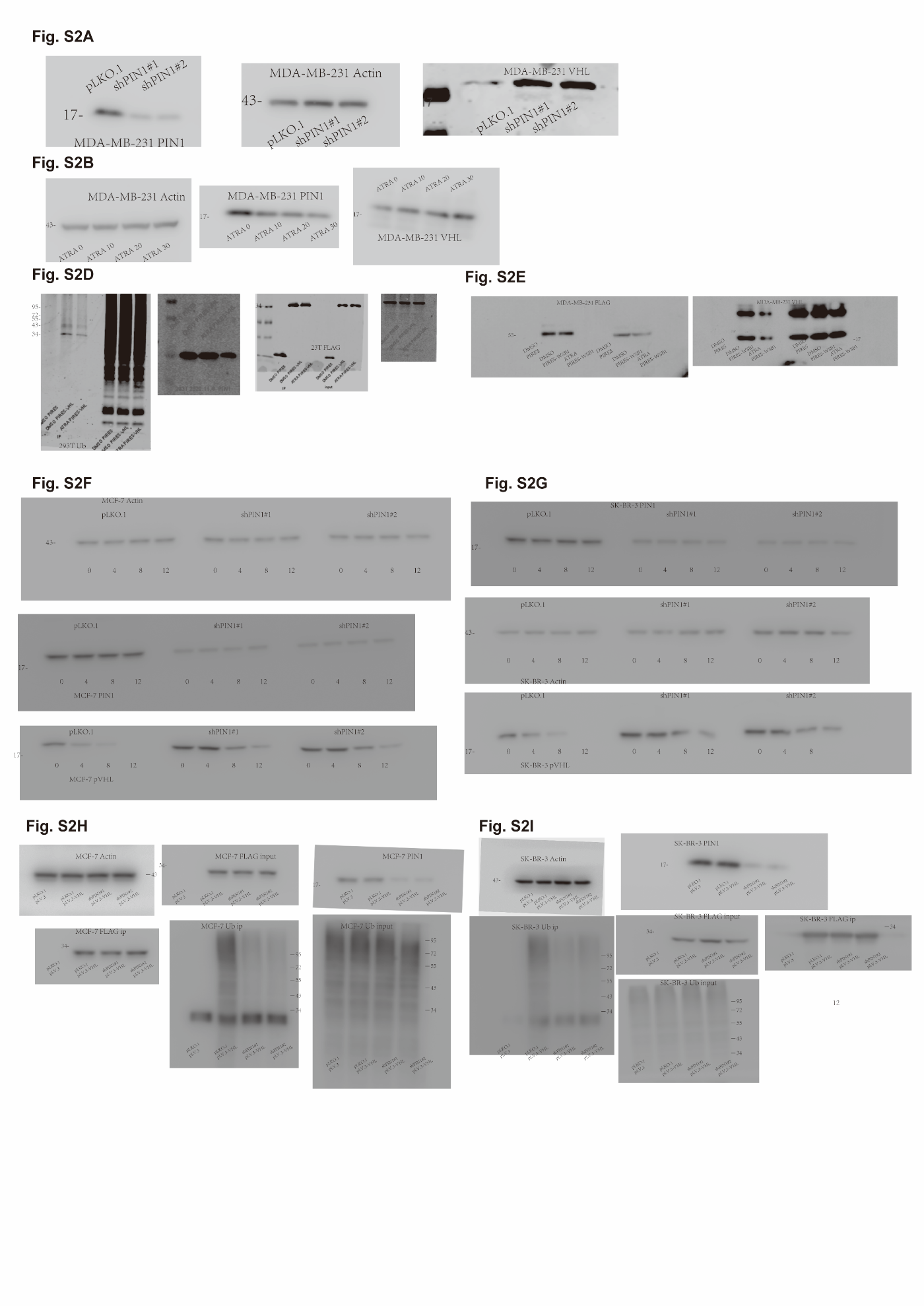


Supplementary Fig. 17: Original scan of the blots presented in the Supplementary Text 1. Related to Supplementary Fig. 2.

Supplementary Fig. 18


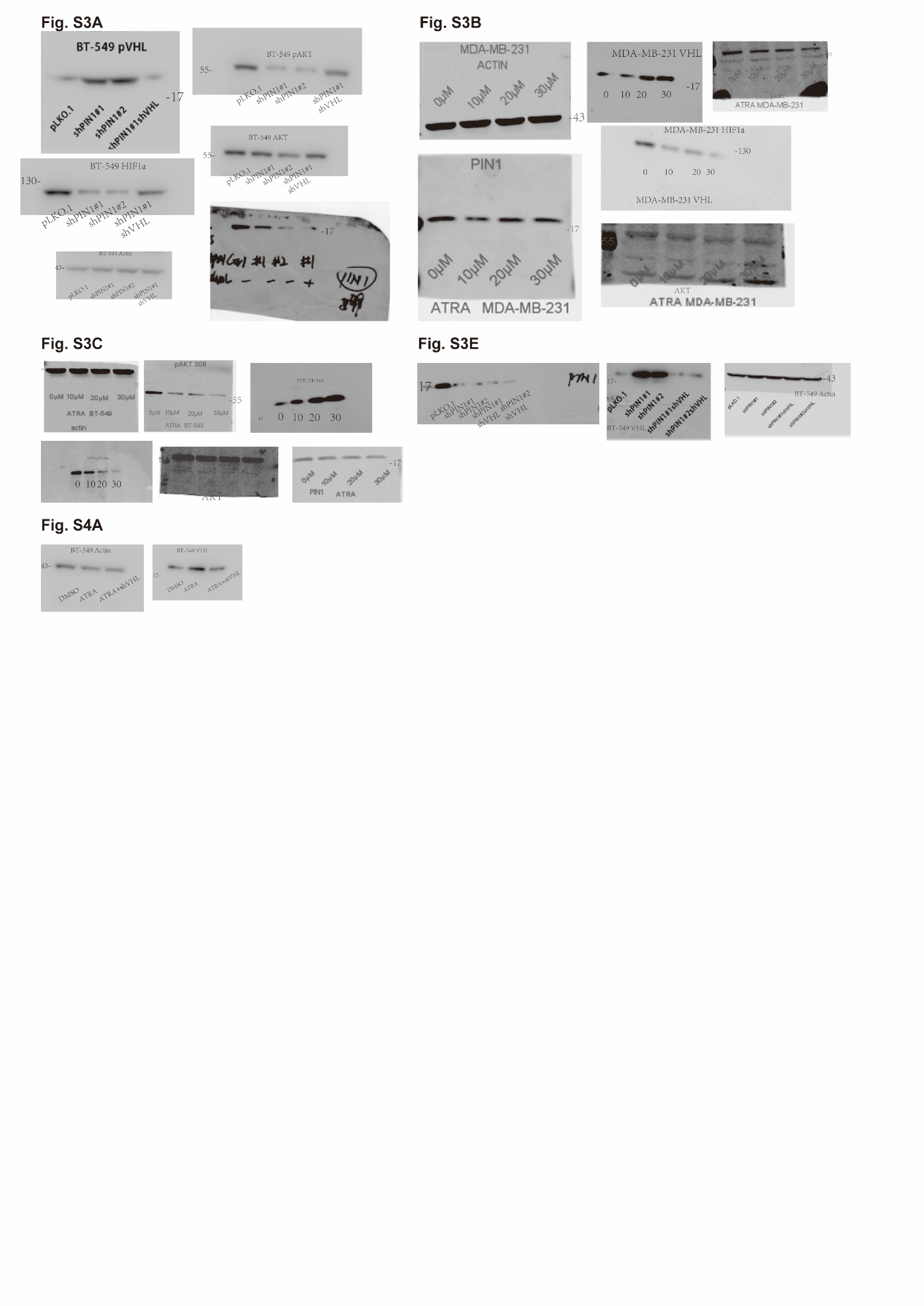


Supplementary Fig. 18: Original scan of the blots presented in the Supplementary Text 1. Related to Supplementary Fig. 3 and Supplementary Fig. 4.

Supplementary Fig. 19


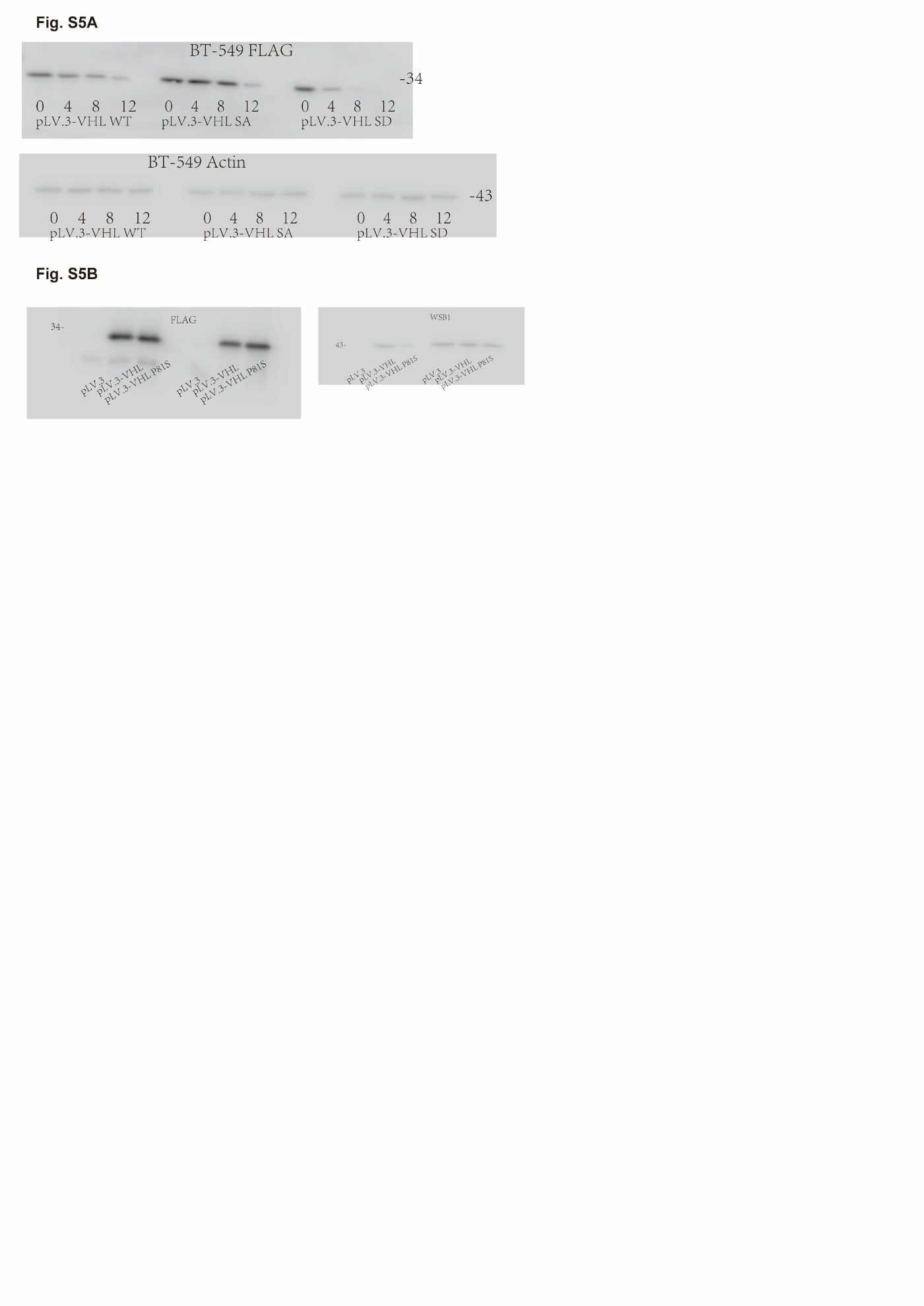


Supplementary Fig. 19: Original scan of the blots presented in the Supplementary Text 1. Related to Supplementary Fig. 5.

Supplementary Fig. 20


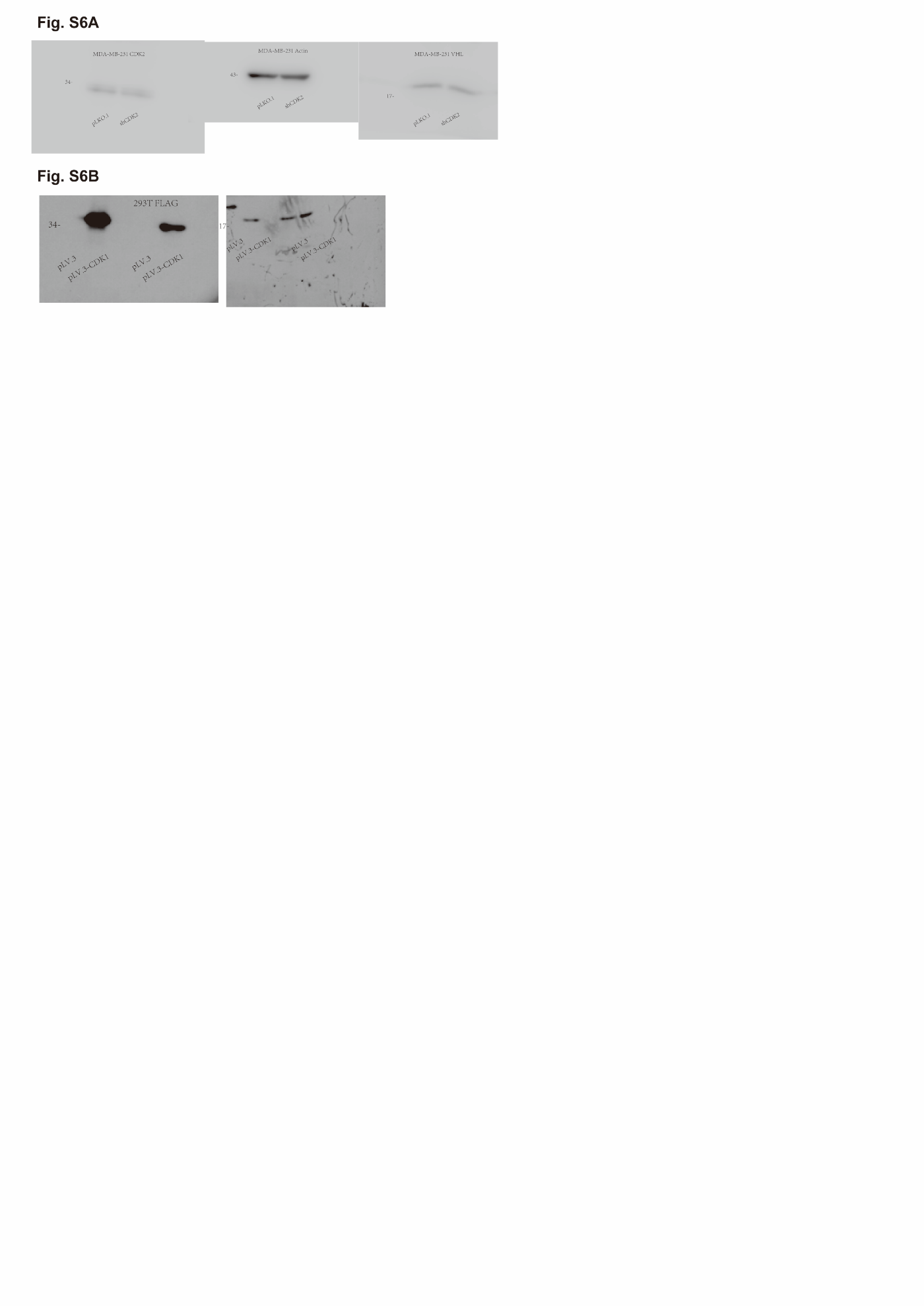


Supplementary Fig. 20: Original scan of the blots presented in the Supplementary Text 1. Related to Supplementary Fig. 6.

Supplementary Fig. 21


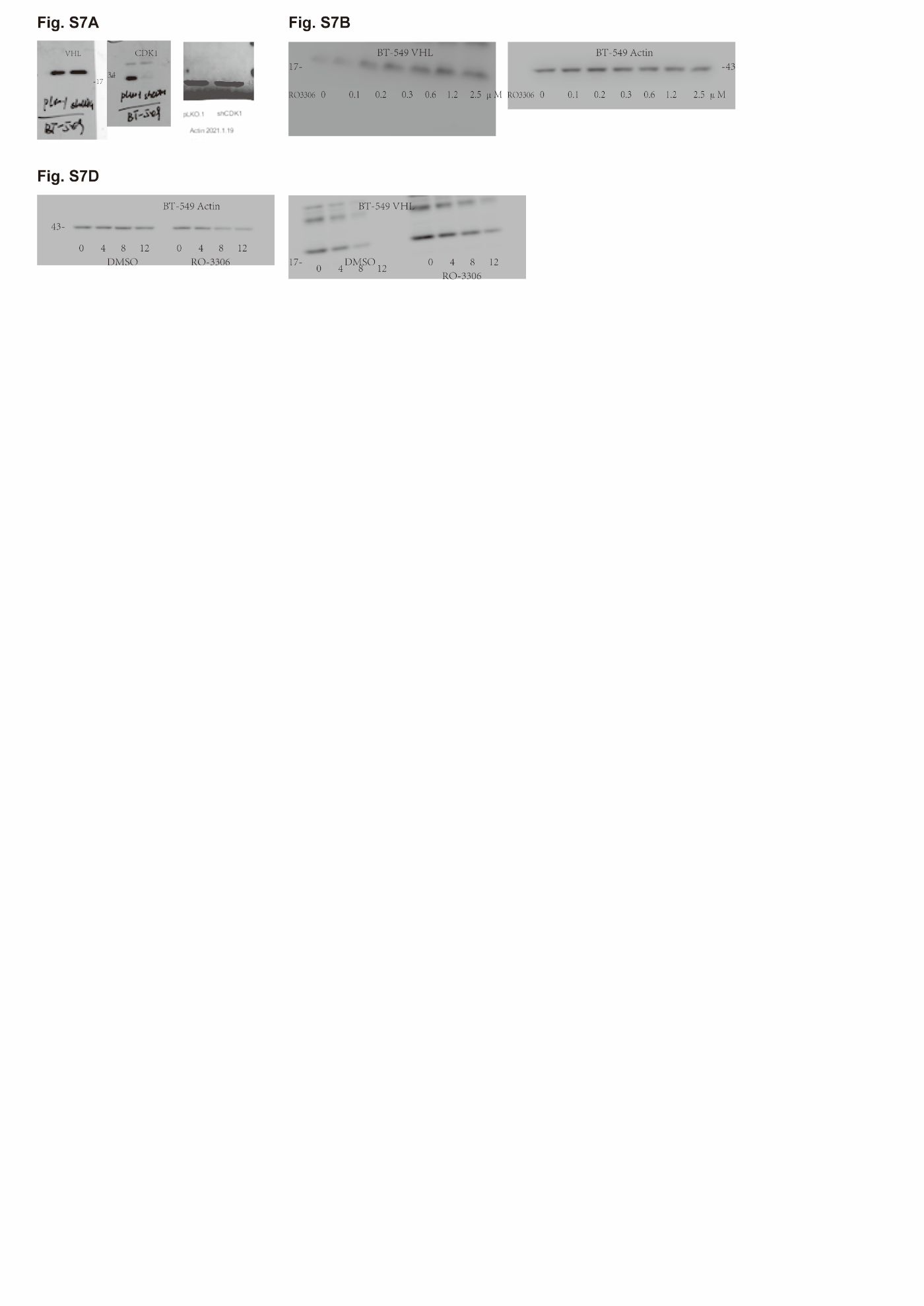


Supplementary Fig. 21: Original scan of the blots presented in the Supplementary Text 1. Related to Supplementary Fig. 7.

Supplementary Fig. 22


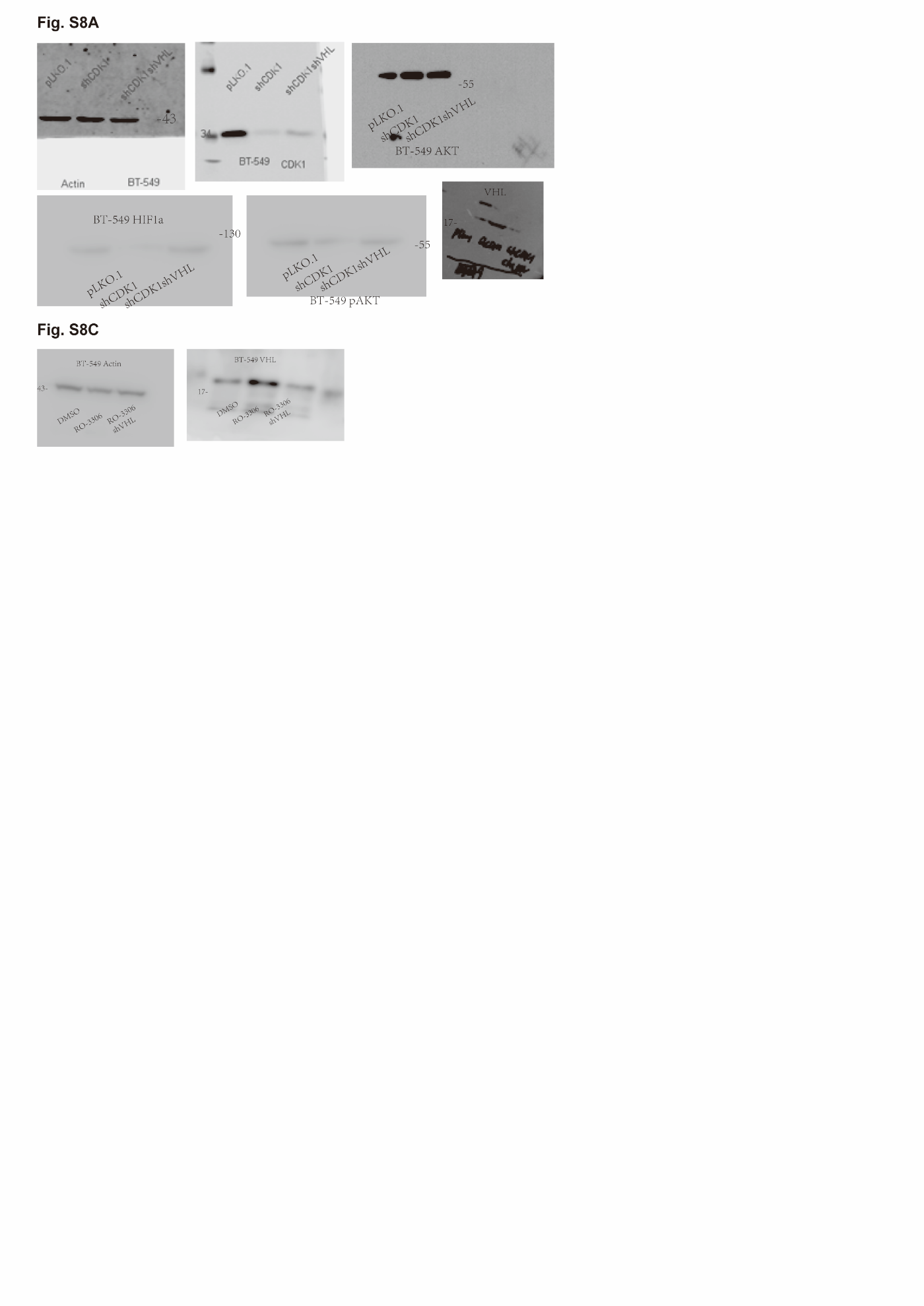


Supplementary Fig. 21: Original scan of the blots presented in the Supplementary Text 1. Related to Supplementary Fig. 8.
